# Model-based joint visualization of multiple compositional omics datasets

**DOI:** 10.1093/nargab/lqaa050

**Published:** 2020-07-21

**Authors:** Stijn Hawinkel, Luc Bijnens, Kim-Anh Lê Cao, Olivier Thas

**Affiliations:** Department of Data Analysis and Mathematical Modelling, Ghent University, 9000 Ghent, Belgium; Quantitative Sciences, Janssen Pharmaceutical companies of Johnson and Johnson, 2340 Beerse, Belgium; Data Science Institute, I-BioStat, Hasselt University, 3500 Hasselt, Belgium; Melbourne Integrative Genomics, School of Mathematics and Statistics, University of Melbourne, 3010 Melbourne, Victoria, Australia; Department of Data Analysis and Mathematical Modelling, Ghent University, 9000 Ghent, Belgium; Data Science Institute, I-BioStat, Hasselt University, 3500 Hasselt, Belgium; National Institute for Applied Statistics Research Australia (NIASRA), University of Wollongong, 2500 Wollongong, New South Wales, Australia

## Abstract

The integration of multiple omics datasets measured on the same samples is a challenging task: data come from heterogeneous sources and vary in signal quality. In addition, some omics data are inherently compositional, e.g. sequence count data. Most integrative methods are limited in their ability to handle covariates, missing values, compositional structure and heteroscedasticity. In this article we introduce a flexible model-based approach to data integration to address these current limitations: COMBI. We combine concepts, such as compositional biplots and log-ratio link functions with latent variable models, and propose an attractive visualization through multiplots to improve interpretation. Using real data examples and simulations, we illustrate and compare our method with other data integration techniques. Our algorithm is available in the R-package *combi*.

## INTRODUCTION

With the latest advances in high-throughput technologies, an increasing number of omics data types is arising that require statistical analysis and data integration tools. These tools must be tailored to the data types under study, whilst being user-friendly with fast computation and easily interpretable results. Here we define data integration as the combination of at least two different types of biological measurements (‘views’) carried out on the same samples. The underlying idea is that their common origin engenders some relationship between the measurements, i.e. the biological state of the organism is reflected from the different views. The goals of such data integration can be very diverse. In this study, we adopt an explorative approach to unearth patterns that extend across different datasets, and identify relationships between features from different views. Using dimension reduction and visualization that focus on the strongest biological patterns in several high-dimensional datasets, our aim is to give researchers a first insight into the data structure and to highlight sample clusters and feature relationships that can be further investigated in follow-up studies.

The simplest way to integrate data is by simply concatenating the different data matrices measured on the same samples, then analyse this data matrix using classical techniques. Its simplicity notwithstanding, the risk is to mix data types that are heterogeneous and thus fail to account for their differences in distribution and noise levels. Other contemporary tools rely on variance partitioning, correlations or other measures based on sums of squares. A common approach is canonical correlation analysis (cca), which finds linear combinations of variables with maximal correlation between views (1), whilst partial least squares (pls) finds linear combinations with maximal covariance (2). These methods, however, can be ill-suited to deal with many types of omics data, such as overdispersed sequence count data. Moreover, these methods rely on the singular value and eigenvalue decompositions that require imputation of missing values and lack flexibility to include covariates, such as patient baseline.

Latent variable models have recently gained traction for the analysis of genomics data (3–8). They are based on the principle that a low number of latent sample variables can capture the most important signals in the dataset. These latent variables are included as components in a regression model, and thus enable great flexibility to specify any outcome distribution and include observed sample-specific variables. Moreover, their estimation naturally handles missing values. When the latent variables are orthogonal, they can be plotted in multiplots to effectively represent the whole model in a single graph (3,5,8).

A popular way to obtain interpretable results from high-dimensional datasets is to enforce *sparsity* during the data integration process (9–11), therefore assuming that only a small fraction of features carry meaningful signal. Usually ℓ_1_-norm penalties are imposed on the feature parameters to set the loadings of many features to zero (e.g. the ‘lasso’). In addition to potential computational difficulties, this approach may struggle with correlated features. In this paper, we address instead the interpretability problem by visualizing features with the strongest signal using thresholding. All features are included in the model, so that loadings of the features with weaker signal can then still be consulted.

Sequence count data quantify the composition of mixtures of nucleic acids, for instance in transcriptomics and microbiomics. The resulting outcomes are integers, but with high variance and many zero observations. As a result, classical count models such as the Poisson and negative binomial distributions do not provide an accurate fit to such data (12–16). However, it is crucial to account for the particular mean-variance structure of sequence count data (17–19). Since the total number of sequences obtained in a particular experiment is mostly unrelated to the original biomass or number of cells, sequence count data are compositional, as are many other types of omics measurements (20). This means that they only contain information on the composition of a nucleic acids mixture (and hence of the specimen under study), and not on its total size or concentration. As a result, changes in one feature affect the proportion of at least another feature. The statistical analysis should take this dependence into account. The most common approach for compositional data is to perform a log-ratio transformation (21), then consider the transformed data as homoscedastic normal. Applying this approach to sequence count data yields to two limitations. Firstly, sequence count data (and count data in general) have a strong mean-variance relationship: features with high means have highly variable counts. The information on these variances is lost in the log-ratio transform. As a result, analysis of the log-ratio transformed counts may be affected by artefacts which are unrelated to the composition, such as library sizes (3). Secondly, sequence count data often have high zero frequencies. This is particularly the case for microbiome and single cell RNA-sequencing data. As logarithms of zero and division by zero are undefined, some imputation is usually applied to replace these zeroes by pseudocounts. An extensive theoretical framework was proposed to impute these zeroes (22), but this imputation results in a data matrix that is a mixture of observed counts and inferred pseudocounts. The uncertainty in the latter is usually ignored in the subsequent analysis. A recent approach proposed to use log-ratio transforms as dedicated link functions for compositional data in a regression model. The parameters are then inverse transformed to obtain the mean model of a composition, rather than transforming the data (23–25). This alternative addresses the two limitations mentioned above, as it allows for the model to be augmented by a suitable count distribution that is appropriate for zero counts and overdispersion. However, this approach has not yet been widely adopted for the analysis of compositional data.

In this work, we combine latent variable modelling and log-ratio link functions with innovative mean-variance modelling to obtain a new model for the integration of multiple views. Our model, called COMBI (Compositional Omics Model-Based Integration) is embedded into a regression framework and can easily incorporate sample-specific variables. Thanks to appropriate parameter restrictions, the final data integration model can be represented graphically in multiplots to facilitate data exploration of multiple views simultaneously. The explicit formulation of the estimating equations unlocks classical diagnostics tools, and allows the model to be fitted on datasets with missing data. We compare our proposed approach with existing approaches, and discuss pros and cons in a simulation study and in real data analysis.

## MATERIALS AND METHODS

### Model description

#### Data structure

Suppose at least two different data matrices or *views*, **X**_(*n* × *p*)_ and **Y**_(*n* × *q*)_ with *p* and *q* features, have been measured on the same source material or individuals (*n* samples). In practice, there is no limit to the number of views that can be included in the analysis, but we explain the concept here with only two matrices. An additional design matrix of sample-specific variables **c**_(*n* × *d*)_ may also be available. Contrary to **X** and **Y**, the elements of **c** are not treated as random variables.

#### Basic latent variable model

The core of our data integration model is based on a set of mean models sharing a set of latent variables, comparable to the *MOFA* model (4):

(1) $$\begin{eqnarray*} g_x\big [E(\mathbf {X}|\mathbf {Z})\big ] = \mathbf {U}_x + \mathbf {Z}\boldsymbol{\Gamma } \end{eqnarray*}$$

(2) $$\begin{eqnarray*} g_y\big [E(\mathbf {Y}|\mathbf {Z})\big ] = \mathbf {U}_y + \mathbf {Z}\boldsymbol{\Theta }, \end{eqnarray*}$$

where g_*x*_ and g_*y*_ are link functions defined according to the data type. $\mathbf {U}_x$ and $\mathbf {U}_y$ are offset matrices that correct for baseline differences, e.g. array intensity or sequencing depths. They define an *independence model* where all samples have an identical composition (i.e. the feature composition is independent of the sample). **Z**_(*n* × *M*)_ is a low dimensional matrix of sample scores on M latent variables and $\boldsymbol{\Gamma }_{(M\times p)}$ and $\boldsymbol{\Theta }_{(M\times q)}$ are view-wise parameter matrices. High values for the sample scores indicate samples that differ strongly from the average sample, whereas large loadings in the parameter matrices indicate features that discriminate between these samples. M is usually set to 2 or 3 in view of making interpretable multiplots (as described below). Restrictions are needed to render this model identifiable. In particular, the latent variables are restricted to be orthogonal: $\mathbf {Z}^T\mathbf {Z}$ = $\text{diag}(\boldsymbol{\psi })$ with diag() defining a diagonal matrix with $\boldsymbol{\psi }$ the non-negative diagonal entries. The coefficient matrices are restricted to be orthonormal: $\boldsymbol{\Gamma }\boldsymbol{\Omega }_x\boldsymbol{\Gamma }^T$ = $\boldsymbol{\Theta }\boldsymbol{\Omega }_y\boldsymbol{\Theta }^T$ = I_*M*_, with $\boldsymbol{\Omega }_x$ and $\boldsymbol{\Omega }_y$ view-specific, diagonal weight matrices (see Supplementary Section 1.1.3) and I_*M*_ the identity matrix of dimension M. Hence our base mean model is identical to *MOFA* but with several substantial improvements: the link functions are better suited for compositional data, the outcome distributions are better suited for sequence count data and the restrictions imposed on the parameters allow to output insightful multiplots to ease interpretation. In addition, since the dimensions are fitted sequentially, the estimates of lower dimensions do not depend on the total number of dimensions required.

#### Sample-specific variables

There are two ways to include sample variables into the analysis, either by considering them as confounders (e.g. batch or sequencing center) and filter out their effect, or by examining them explicitly and interpret their biological relationship (a constrained analysis) (3). In the case of confounding variables, their effect is eliminated by conditioning on them prior to the estimation of the latent variables. Let **R** and **S** denote the design matrices of the confounding variables in views **X** and **Y**. We define the following model:

(3) $$\begin{eqnarray*} g_x\big [E(\mathbf {X}|\mathbf {Z}, \mathbf {R})\big ] = \mathbf {U}_x + \mathbf {R}\boldsymbol{\Phi } + \mathbf {Z}\boldsymbol{\Gamma } \end{eqnarray*}$$

(4) $$\begin{eqnarray*} g_y\big [E(\mathbf {Y}|\mathbf {Z}, \mathbf {S})\big ] = \mathbf {U}_y + \mathbf {S}\boldsymbol{\Xi } + \mathbf {Z}\boldsymbol{\Theta }. \end{eqnarray*}$$

where $\boldsymbol{\Phi }$ and $\boldsymbol{\Xi }$ are parameter matrices. The estimates of $\mathbf {Z}$, $\boldsymbol{\Phi }$ and $\boldsymbol{\Gamma }$ will then be free of the effect of the confounders.

In case of a constrained analysis, the sample scores in **Z** are no longer unrestricted, but become metavariables that are linear combinations of the observed sample variables. In particular $\mathbf {Z} = \mathbf {c}\boldsymbol{\Lambda }$, where $\boldsymbol{\Lambda }$ is a *d* × *M* matrix with the *environmental gradients* (3,26) in the columns. Each environmental gradient consists of loadings that reflect the importance of the sample variables in shaping both views and their relationship with the features. The gradients are restricted to be orthonormal: $\boldsymbol{\Lambda }^t\boldsymbol{\Lambda } = {\bf I}_M$. If the design includes categorical variables, then **c** will be constructed with dummy (indicator) variables for every level without using a reference level. The elements of $\boldsymbol{\Lambda }$ corresponding to the dummy variables of the same categorical variable are restricted to have zero sum. This arrangement avoids dependence on the choice of reference variable in view of the normalization restriction above. It also leads to informative plots with all levels of the categorical variables shown, without hidden reference levels. The continuous variables in **c** are normalized to have standard deviation 1.

#### Compositionality

The link function determines the range of the expected outcomes of a regression model. For compositional views, we choose the centered log-ratio transform (clr) as a link function, which is defined as:

(5) $$\begin{equation*} \text{clr}(\mathbf {x}) = \log \Bigg (\frac{x_1}{(\prod _{j=1}^p x_j)^{1/p}}, ..., \frac{x_p}{(\prod _{j=1}^p x_j)^{1/p}}\Bigg ). \end{equation*}$$

The inverse transformation (clr^−1^, also known as the *softmax*) is defined as:

(6) $$\begin{equation*} \text{clr}^{-1}(\mathbf {x}) = \Bigg (\frac{\exp (x_1)}{\sum _{j=1}^p \exp (x_j)}, ..., \frac{\exp (x_p)}{\sum _{j=1}^p \exp (x_j)}\Bigg ). \end{equation*}$$

The result of this inverse transformation is a vector with values between 0 and 1 that sum to 1, i.e. a *composition*. The advantage of the clr over other log-ratio transforms is that it allocates a single parameter to each feature, which is a crucial property for making interpretable biplots (25,27). The mean model for the outcome of feature *j* in sample *i* is then:

(7) $$\begin{eqnarray*} E(X_{ij}|\mathbf {Z}_{i}) &=& \Big [\text{clr}^{-1}(\mathbf {u}_x + \mathbf {Z}_{i}^t\boldsymbol{\Gamma })\Big ]_js_i \nonumber \\ &=& {\pi _{ij}(\boldsymbol{\pi }^{{\rm indep}}, \mathbf {Z}_{i}, \boldsymbol{\Gamma })s_i}, \end{eqnarray*}$$

with s_*i*_ an estimate of the baseline sample intensity in sample *i* (e.g. the sequencing depth), $\boldsymbol{\pi }^{{\rm indep}} = \text{clr}^{-1}(\mathbf {u}_x)$ the proportion vector under the independence model and $\pi _{ij}(\boldsymbol{\pi }^{{\rm indep}}, \mathbf {Z}_{i}, \boldsymbol{\Gamma })$ the *j*-th feature proportion under the full model. This model can be regarded as follows: each dimension *m* ‘perturbs’ the lower dimensional composition of sample *i* with direction $\boldsymbol{\Gamma }_m$ and strength $\mathbf {Z}_{im}$ to form a new composition, which also sums to 1 (23,24). The perturbation operator ⊕ is defined for a composition **u** and a strictly positive vector **v** as proposed by Aitchison (21):

(8) $$\begin{equation*} \mathbf {u} {\oplus } \mathbf {v} = \frac{1}{\sum _{j=1}^p u_jv_j}\Big (u_1v_1, ..., u_pv_p \Big ). \end{equation*}$$

The composition of sample *i* is then (23):

(9) $$\begin{equation*} \boldsymbol{\pi }_i = \text{clr}^{-1}(\mathbf {u_x}) {\oplus } \text{clr}^{-1}(\boldsymbol{\Gamma }_1)^{\mathbf {Z}_{i1}} {\oplus } \cdots {\oplus } \text{clr}^{-1}(\boldsymbol{\Gamma }_M)^{\mathbf {Z}_{iM}}. \end{equation*}$$

So far we have only specified mean models. To allow these models to be estimated, more information is needed on the outcome distributions. This can be done by specifying a parametric distribution, or by specifying a variance model as described in the next subsection.

#### Quasi likelihood estimation for sequence count data

Count distributions that are appropriate for sequence count data are not available, hence we chose to estimate the parameters of these views through quasi-likelihood. Quasi-likelihood is a semiparametric estimation technique, whereby only the mean and the variance of an outcome are modelled, and the higher moments are left unspecified (28). The estimating equations for κ have the following general form

(10) $$\begin{equation*} \sum _{i=1}^n \frac{\partial E(X_{ij})}{\partial \kappa }\frac{X_{ij}-E(X_{ij})}{V\big [E(X_{ij})\big ]} = 0, \end{equation*}$$

with κ some parameter that is part of the mean model and *V*[*E*(*X*_*ij*_)]∝Var(*X*_*ij*_). Intuitively, observations with a large raw residual, a low variance and whose expectation varies strongly with the parameter have a strong influence on the parameter estimate. In some special cases of *V*[*E*(*X*_*ij*_)], these estimating equations correspond to score equations from maximum likelihood estimation, but in general there is no underlying likelihood function being maximized by solving these equations.

The model for the variance in sequence count data can be inspired by a parametric assumption, e.g. assuming the variance equal to the mean as for the Poisson distribution. Alternatively, the mean-variance trend can be estimated non-parametrically from the data, as is often done in genomics (17–19). Yet, as sequence count data are compositional, we are modelling mean compositions and treat the sequencing depths as ancillary statistics. Therefore we chose to model the trend between the mean relative abundance and the variance for every dimension *m* (henceforth called the ‘abundance-variance trend’ $v$_*m*_), and include the sequencing depth only as a constant. More formally we assume that

(11) $$\begin{eqnarray*} && \text{Var}(X_{ij}|\pi _j^{{\rm indep}}, \mathbf {Z}_{.1,m}, \boldsymbol{\Gamma }_{1,m.}, s_i) \nonumber \\ && \quad = v_m\left(\pi _{ijm}(\boldsymbol{\pi }^{{\rm indep}}, \mathbf {Z}_{.1,m}, \boldsymbol{\Gamma }_{1,m.})\right)s_i, \end{eqnarray*}$$

where $\mathbf {Z}_{.1,m}$ indicates the first *m* columns of $\mathbf {Z}$ and $\boldsymbol{\Gamma }_{1,m.}$ the first *m* rows of $\boldsymbol{\Gamma }$. The variance is thus calculated conditional on the first *m* dimensions of the model fitted so far.

The smooth function $v$_*m*_ needs to be estimated from the data, based on the relationship over all features between the relative abundances under the independence model $\boldsymbol{\pi }^{{\rm indep}}$ and the feature-wise weighted variances of under the model of dimension *m*. The latter are estimated as

(12) $$\begin{equation*} \begin{aligned} \overline{\text{Var}(X_j| \mathbf {Z}_{.1,m})} = \frac{\sum _{i=1}^n\frac{\big (X_{ij} - E(X_{ij}|\mathbf {Z}_{.1,m})\big )^2}{s_i}}{n-1}. \end{aligned} \end{equation*}$$

**Figure 1.**
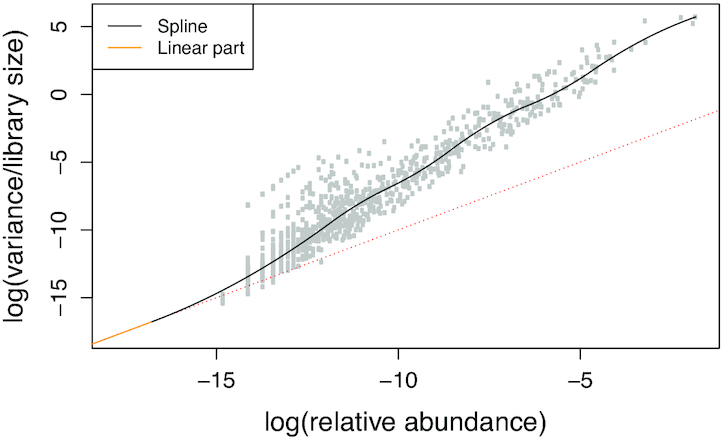
Abundance-variance trend (in black) as estimated from the HMP2 microbiome dataset under the independence model. The red dashed line corresponds to a variance proportional to the mean relative abundance. The linear line in orange reflects the heuristic that the variance can never drop below the mean.

A cubic smoothing spline on the log-scale is chosen for $v$_*m*_. As a heuristic, it is restricted to coincide with the diagonal line for low abundances, which corresponds to the variance model of the Poisson distribution (17,29) (detailed in Supplementary Section 1.2.3). Figure 1 shows an example of such abundance-variance trend in microbiome data. Given an estimated sequencing depth *s*_*i*_ and a modelled feature proportion π_*ij*_, the predicted variance $v$_*m*_(π_*ij*_)*s*_*i*_ is then inserted into (10). Of course, $\boldsymbol{\pi }^{indep}$ remains constant, but $\overline{\text{Var}(X_j|\mathbf {Z}_{.1,m})}$ changes as the model is fitted, such that the abundance-variance trend $v$_*m*_ needs to be iteratively re-estimated for each dimension *m*.

#### Continuous data

For modelling other data types with approximate (log-)normal distributions (e.g. microarray), we mainly follow the tracks of the popular *limma* package (30). The data may be log-transformed, and is then modelled using a simple linear model with identity link. The estimates of the feature-wise variances are shrunken towards a common value using an empirical Bayes procedure (31).

#### Missing data

Missing data are a common issue in genomics data. However, in data integration the problem is exacerbated as measurements may be missing for some subjects in one view and for other subjects in another view. This problem is often tackled by removing subjects for whom information is not available for all views (a ‘complete cases’ analysis). Yet this approach throws away useful data, and is only valid under the missing completely at random assumption. Another strategy is to impute missing measurements from available data, but this adds complexity to the method and may affect the results. Model-based methods can naturally deal with missing observations by excluding missing values from the estimating equations. For example, in Equation (10), index *i* only runs over observations that are not missing. Such approach is advantageous, as it optimally extracts the information from a given dataset without the need for omission or imputation of data, and valid when data are missing at random (MAR). However, the approach is not valid when values are not missing at random (MNAR or informative missingness).

#### Influence functions

Noise levels may vary considerably between different views, as different technologies are designed to measure distinct biological processes. Once the data integration model has been fitted, it may be interesting to examine which datasets contributed most to the estimation of the latent variables or the environmental gradient. Because the estimating equations are stated explicitly, these contributions can be extracted directly through their corresponding influence functions. Figure 4 illustrates how influence functions can reveal the views that contribute most to the estimation of the different model components; see Supplementary Section 1.3 for an exhaustive discussion.

### Construction and interpretation of multiplots

Once fitted, the low dimensional mean model can be plotted in an integrated graph. First, the scores of the latent variables are plotted as dots in two (or three) dimensions, i.e. the pair (z_*i*1_, z_*i*2_) determines the location of sample *i* (1,$\ldots$, n). The distances between those dots reflect the dissimilarities between the samples across all views. For a constrained integration, the loadings $\boldsymbol{\lambda }$ are added as labels at locations (λ_*k*1_, λ_*k*2_), (*k* = 1,$\ldots ,d$). Their distance from the origin reflects the importance of sample-specific variable *k* in explaining the variability across all views. However, note that no direct interpretation of their distances to the sample locations is available, and that distances between variable labels are not meaningful either. Finally, the feature loadings in $\boldsymbol{\Gamma }$ and $\boldsymbol{\Theta }$ of the different views are added as labels. Because of the high dimensionality, one may choose to plot features with the largest loadings only (i.e. furthest away from the origin) to avoid overplotting (‘thresholding’). The interpretation of these feature labels depends on whether the dataset is compositional.

For non-compositional data types, the interpretation of the loading vector $\boldsymbol{\gamma }_{a}$ for a given feature *a* is simple: feature *a* has a higher mean than in the average sample in a sample *i* that lies on the same side of the origin (i.e. $\boldsymbol{\gamma }_{a}^t\mathbf {Z}_{i}>0$), and a lower mean otherwise. The outcomes of these features are also positively associated with the sample variable *k* whose label lies on the same side of the origin as the feature label (i.e. $\boldsymbol{\gamma }_{a}^t\boldsymbol{\lambda }_{k}>0$). Moreover, two features plotted on the same side of the origin are positively correlated, whilst features on opposite sides indicate a negative correlation, regardless of their respective views.

**Figure 2.**
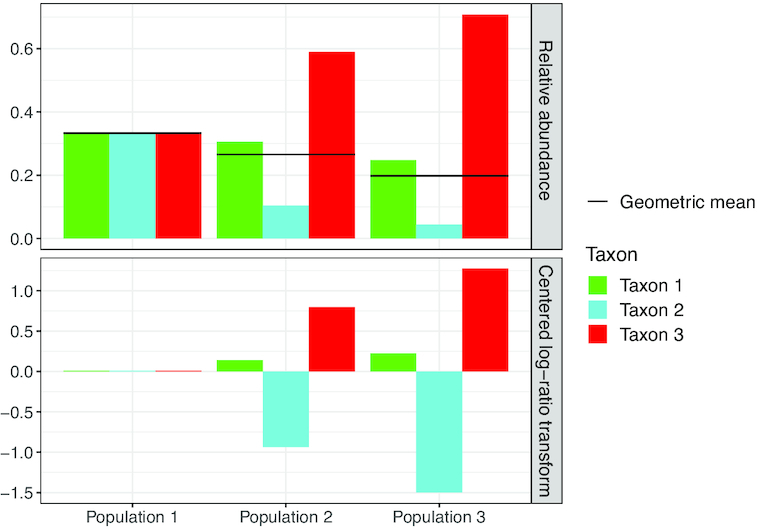
Toy example illustrating the difficulties in interpreting the centered log ratio (clr) transform. In the top panel, three toy populations of three taxa are shown, varying from even on the left to uneven on the right. Horizontal lines represent corresponding geometric means of the relative abundances. The bottom panels shows the clr transforms of these populations. Notice how taxon 1 decreases in abundance from left to right, whilst its clr transform increases, because the geometric mean drops faster than the relative abundance of taxon 1 as the population becomes less even.

Interpretations involving features from compositional datasets (i.e. compositional biplots as introduced by Aitchison and Greenacre (27)) are less straightforward, since compositionality imposes a certain dependence between features. Hence, feature loadings should not be interpreted individually, but always in combination with at least one other feature. A mathematically convenient quantity is the log-ratio of the feature proportion to the geometric mean (gm) of all feature proportions: $\log \left(\frac{\pi _j}{\text{gm}(\boldsymbol{\pi })}\right)$. When the feature label lies for instance on the same side of the origin as the sample, this means that the log-ratio of this feature is larger in this sample than in the average sample. Yet this may not be very meaningful biologically, because the geometric mean of feature proportions may be a very intractable summary. The geometric mean can be regarded as a measure of evenness, similar to the Shannon index (32). It is maximal (equal to 1/*p*) for a perfectly even composition, but decreases irregularly as the composition departs from perfect evenness. As a result, despite the fact that the log-ratio evolves linearly with the latent variables, the feature proportion often varies non-monotonically. These capricious effects are demonstrated in Figure 2.

Therefore, we consider ratios of two (or more) features (27). Denoting $\boldsymbol{\gamma }_b$ the feature loading of feature *b*, we consider:

(13) $$\begin{equation*} \log \left(\frac{\pi _{ia}}{\pi _{ib}}\right) - \log \left(\frac{\pi _a^{{\rm indep}}}{\pi _b^{{\rm indep}}}\right) = \mathbf {Z}_i^t(\boldsymbol{\gamma }_{a}-\boldsymbol{\gamma }_{b}). \end{equation*}$$

**Figure 3.**
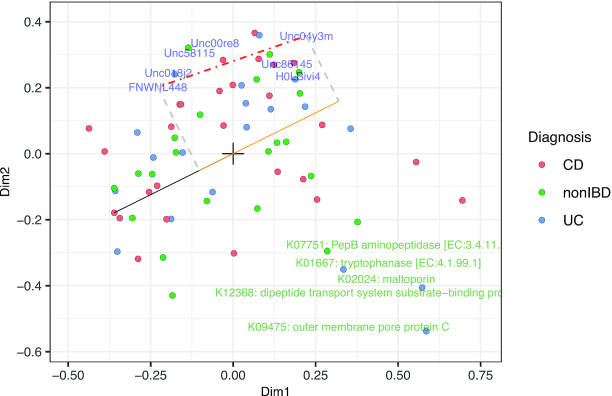
Data integration triplot of the microbiome and proteome datasets from the HMP2 project. Coloured dots represent patients, labels represent features of microbiome (blue) and proteome (green). We focus our interpretation on the link between taxa *Unc04y3m* and *FNWNL488* (shown with a red dashed line). The black line connects the CD sample on the left with the origin, and the dashed grey lines illustrate the projection of the taxa *Unc04y3m* and *FNWNL488* link onto this black line; the projection itself is shown as an orange line. This projection is large, implying that the ratio FNWNL448/Unc04y3m is much larger in this sample than in the average sample. On the contrary, taxa *Unc018j2* and *FNWNL488* lie close together in the top left corner, and have a short link. This implies that their ratio differs very little over all samples, and these features’ abundances are thus correlated.

Note that the geometric mean has been eliminated from the expression. The difference $(\boldsymbol{\gamma }_{a}-\boldsymbol{\gamma }_{b})$ between vectors is known as the *link* in a plot, i.e. the straight line connecting the points defined by $\boldsymbol{\gamma }_a$ and $\boldsymbol{\gamma }_b$. This difference is small when the features labels are close on the multiplot (i.e. the Euclidean distance between the loadings is small). In that case, this means that for *any* sample, the ratio of the relative abundances $\frac{\pi _{ia}}{\pi _{ib}}$ and the ratio of the relative abundances under the independence model $\frac{\pi _a^{{\rm indep}}}{\pi _b^{{\rm indep}}}$ do not differ by much. In a compositional setting, a stable ratio across samples indicates that the features are strongly correlated (21). When this link is large, the projection of the latent variable scores $\mathbf {Z}_{i}$ onto the link (i.e. $\mathbf {Z}_{i}^t(\boldsymbol{\gamma }_{a}-\boldsymbol{\gamma }_{b})$) indicates how much and in which direction the ratio $\frac{\pi _a}{\pi _b}$ differs from that under the independence model in sample *i* (27). This implies that features lying on the same radius from the origin but far apart (i.e. we have $\frac{\boldsymbol{\gamma }_{a}^t\boldsymbol{\gamma }_b}{||\boldsymbol{\gamma }_{a}||||\boldsymbol{\gamma }_b||}\approx 1$ but $||\boldsymbol{\gamma }_{a}|| \ne ||\boldsymbol{\gamma }_{b}||$) are not necessarily strongly correlated in all samples! These interpretations are illustrated in Figure 3. The interpretation of combinations of features from different compositional views is very difficult.

#### Importance of the dimensions

Many ordination methods output measures of importance of the dimensions, typically as the fraction of total variability. Yet this is difficult for many non-normal data types, and hard to compare across different views. Moreover, as part of the variability in any stochastic dataset is noise, it unknown which fraction of the total variability this noise represents, and hence hard to know which fraction of total variability the ordination should strive to explain. For these reasons, our COMBI method does not yield any measures of variability explained by the fractions, but the axes are forced to be square. As a result, the euclidean distances between sample dots truthfully reflect dissimilarities between the samples, and a large spread of the sample scores in one dimension indicates a large variability in this dimension.

### Real case studies and analysis

We considered three studies. The Human Microbiome Project 2 (HMP2), or integrative HMP (iHMP), aimed to investigate the relationship between the microbiome and host responses. One branch focused on healthy and IBD patients (with either Crohn’s disease (CD) or ulcerative colitis (UC)) (33), which constitutes our first study. The second study is of Zhang et al. (34) who investigated the effect of pulsed antibiotic treatments (PAT) on the onset of Type I diabetes (T1D) in mice. The gut microbiome composition, as well as many host genomics measurements were recorded over time. We refer to this study as the Zhang data. A third study (the Gavin data) contain microbiome data and human and microbial proteome data from patients with T1D, as well as from healthy controls (35).

#### Real data analysis

All datasets were subjected to unconstrained analysis with default settings of the *combi* package. For the HMP2 package, all unconstrained two-way integrations of the microbiome, proteome and virome datasets were fitted, as well as a three-way integration. Constrained integrations were fitted on the same dataset using the ‘biopsy location’, ‘diagnosis’ and ’sex’ variables. On the Zhang data, the two-way integrations of microbiome-metabolome and microbiome-immunological data were fitted. Constrained integrations were fitted on the same dataset using the ‘Treatment’ (PAT), ‘Sex’, ‘Time’ and ‘Sample.Weight’ variables. On the Gavin data the unconstrained and constrained three-way integrations of microbiome and human and microbial proteome data were fitted, the constrained integration using the ‘T1D status’, ‘age’, ‘Number of auto antibodies’ (abnoNum), ‘disease duration’ and ‘hba1c’ variables. All datasets were treated as compositional and modelled using quasi-likelihood, except for the Zhang metabolome and Gavin proteome data, for which Gaussian models were fit without compositionality constraint.

### Simulation study

#### Data generation

To evaluate and benchmark the performance of our method, data were generated according to three different paradigms. The first data generation strategy assumes that the sequence count data follow the negative binomial distribution. For metabolome and Gavin proteome data, a Gaussian distribution is assumed. The parameters of these distributions were estimated from the real datasets through maximum likelihood. Parameter values were then sampled from this pool of parameter estimates, and random data were drawn from the corresponding distributions. The samples were split into two equally sized groups, and for 10% of the features, a fold change was introduced in one of the groups. For compositional data this happened both with and without compensation. Compensation means that the abundance of some of the features is increased and for others the abundance is decreased, such that the abundance of the remaining features is left unaltered (12). For Gaussian data the fold change was 0.1, for sequence count data it was 4. In the second strategy, data were generated using the *SimSeq* procedure (14). The IBD status was used as grouping variable for the HMP2 data, the treatment group for the Zhang data, and the T1D status for the Gavin data. The same samples were used to draw observations for both views, in order to preserve correlations between views. In the third strategy, real data were reshuffled by permuting the samples of different views independently. This breaks the correlation between features from the different views, which provides a useful null setting with real data characteristics. In all cases, the number of samples was *n* = 40, and only the *P* = 1000 most abundant features were used. In each setting, 100 Monte-Carlo runs were executed.

#### Benchmark methods

We compared our method with following other integration methods or approaches. JIVE and MOFA were run using the *r*.*jive* (36) and *MOFA* (37) packages, respectively. Canonical correlation analysis was applied both with and without shrinkage, and with and without prior clr transformation, using the *PMA* package (38). We also considered concatenating the view matrices by row to perform principal component analysis to either the raw or clr-transformed data, as well as to perform correspondence analysis on the raw data. Partial least squares with canonical mode was applied to raw and clr-transformed data as implemented in the *mixOmics* package (39). Prior to all clr transformations, zero counts were imputed using the *cmultRepl*() function in the *zCompositions* package (40). Unless mentioned otherwise, default settings were used for all packages, with two dimensions. All analyses were run in R programming language, version 3.6.1 (41). Details on the software and package versions used can be found in the Supplementary Section 6.

#### Method evaluation

The methods were evaluated based on the correlation of the sample scores with the sample-wise sums of each view separately and with the overall sum. To quantify how well the methods identify correlated features, the inner products of all feature loadings were calculated, and the Wilcoxon rank sum test was performed to assess whether truly correlated features had a higher inner product than uncorrelated taxa. We choose the rank approximation because only the features with the strongest signal will be plotted, and hence their correct ranking is crucial. The standardized Wilcoxon rank sum test statistic was then used as a measure of discrimination between correlated and non-correlated taxa. This test statistic was calculated for all feature combinations as well as for between-view combinations only. The pseudo-F statistic was used to evaluate the clustering of samples from the same group (3,42).

## RESULTS

### Real data results

We summarize in this section the most important findings; the remainder of the analyses are presented in the Supplementary Material.

#### HMP2 data

**Figure 4.**
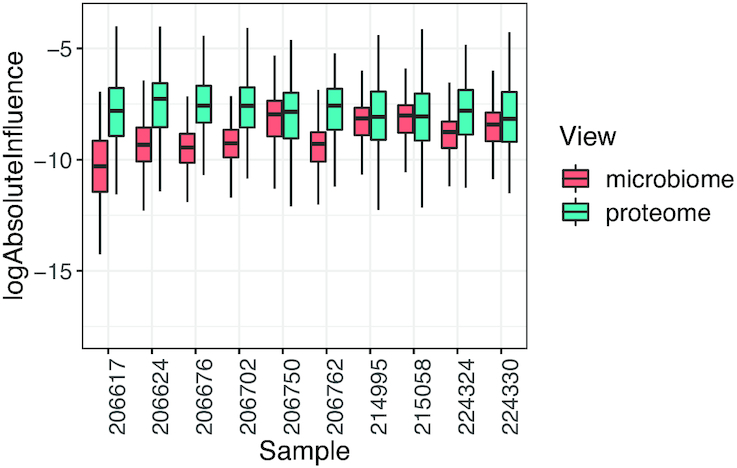
Data integration of the microbiome and proteome datasets from the HMP2 project. Boxplots of log absolute influence on the estimation of the latent variables of the first 10 samples in dimension 1.

**Figure 5.**
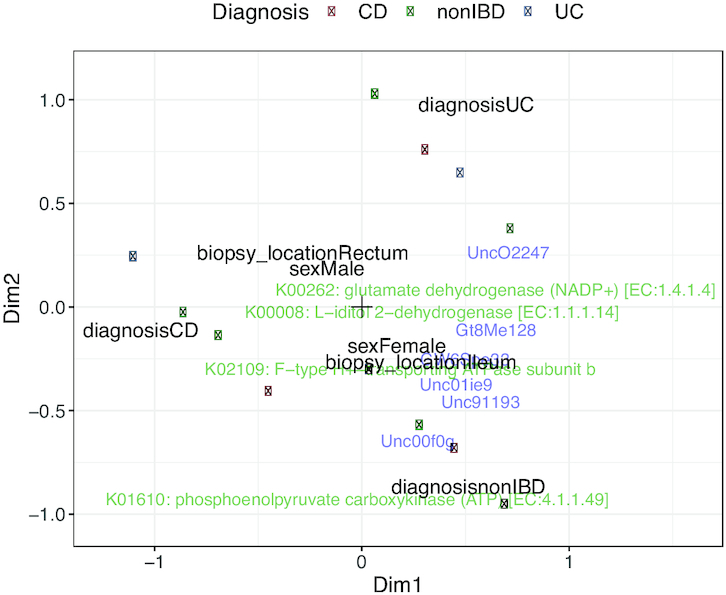
Constrained ordination of the microbiome and proteome datasets from the HMP2 project. Coloured dots represent patients, labels represent features of microbiome (blue) and proteome (green). Black labels represent patient variables.

The unconstrained integration of HMP2 microbiome and proteome datasets is shown in Figure 3. No clear clusters of patients with different disease statuses are visible, as the variability in this dataset is large. The influence plot in Figure 4 reveals that the proteome view has the largest influence on the estimation of the sample variables. Yet, the constrained ordination of the same data (Figure 5) identifies the disease status as more important in driving variability than gender or biopsy location.

#### Zhang data

**Figure 6.**
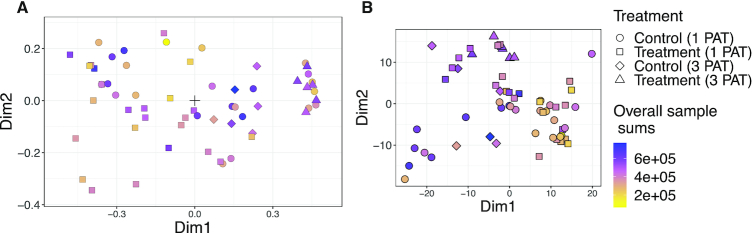
Unconstrained sample ordination of Zhang microbiome and immunological data through (A) our COMBI method and (B) PCA on clr-transformed data. Colours reflect overall sample sums, shapes represent T1D status: control (PATCON) or treatment group (PAT) in experiment with 1 (1 PAT) or 3 (3 PAT) pulsed antibiotic treatments.

The unconstrained integration of the Zhang microbiome and immunological data (Figure 6A and Supplementary Figure S11) reveals that in these two views, the variability is much larger in the single PAT than in the triple PAT group. For comparison, the sample ordination by PCA with clr-transform is shown in Figure 6B. In this ordination, the separation of the different treatment groups is less clear, as the effect of the overall sample sum distorts the ordination.

**Figure 7.**
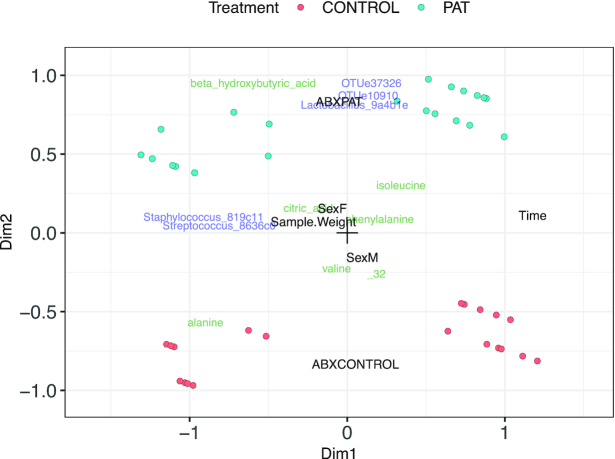
Quadriplot of the constrained integration of Zhang microbiome and metabolomics data. Coloured dots represent mice, blue and green labels are microbial taxa and metabolites, respectively. Black labels represent components of the environmental gradient.

The constrained ordination of the microbiome and metabolomics data in Figure 7 shows that time is the most important sample variable in driving variability, followed by antibiotics treatment group. Citric acid, isoleucine and valine can be seen to be associated to the antibiotics treatment, as was also discovered by the authors of the original study (34) and can be seen in the unconstrained integration (Supplementary Figure S15) as well.

#### Gavin data

**Figure 8.**
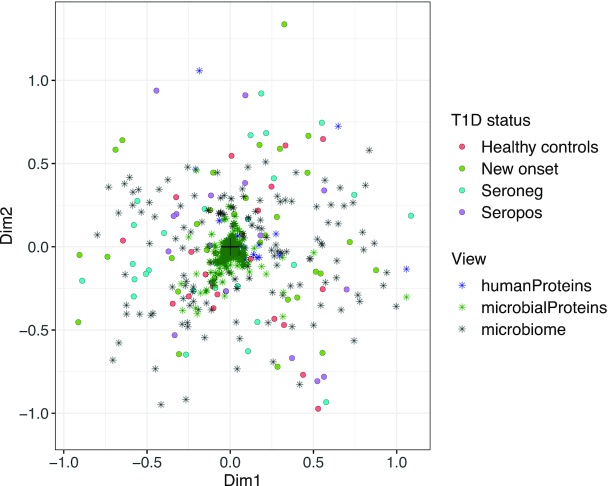
Unconstrained integration of Gavin microbiome and human and microbial proteome data. Dots represent samples, stars represent all features of the different views, without thresholding.

**Figure 9.**
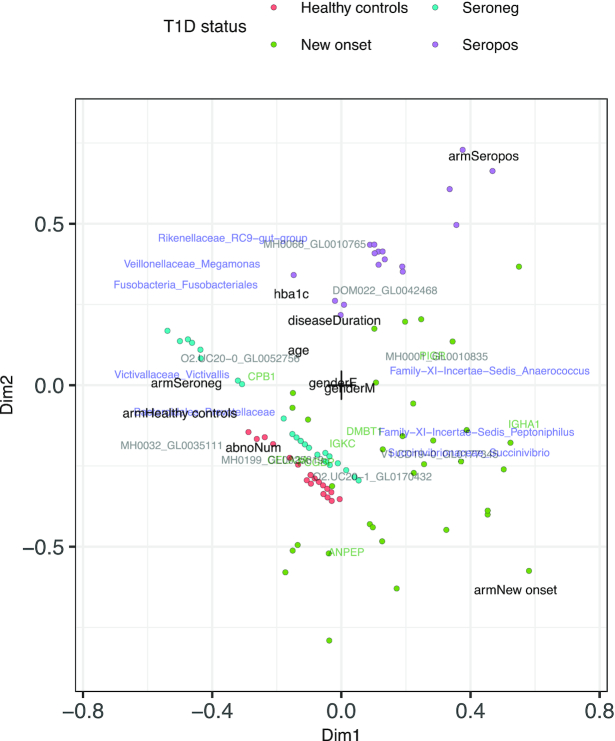
Constrained integration of Gavin microbiome and human and microbial proteome data. Blue labels represent taxa, red labels microbial proteins and green labels human proteins. Black labels represent components of the environmental gradient. Healthy and seronegative states are similar, as expected since they represent similar physiological states.

The unconstrained ordination of the Gavin data (Figure 8) did not show any clear sample clusters, as noise levels are too high. The constrained integration of the Gavin data (Figure 9 and Supplementary Figure S17) confirm that the healthy control and seronegative statuses are most similar. Apart from T1D status, we noted hba1c (a measure for average past blood glucose levels), disease duration and number of auto antibodies (abnoNum) as strong drivers of variability in proteome and microbiome, whereas gender was unimportant. The *PIGR*, *IGKC* and *IGHA1* human proteins are markers of inflammation that are more abundant in seropositive and diseased patients, which may point to abnormal immune response in their gut. Chymotrypsin Like Elastase 3A (*CELA3A*) was higher in abundance in healthy and seronegative patients, as was also found by the authors of the original study.

### Simulation results

In parametric as well as non-parametric simulations, the sample scores of PCA (with and without clr-transformation) and MOFA were correlated with the library sizes in some scenarios (see Figure 10; Supplementary Figures S12–14 and 18–35), confirming our observation from the real case study in Figure 6. Methods based on covariances (cca and pls) were best at identifying correlated taxa, both within and between datasets (see Figure 11 and Supplementary Figure S36). These methods perform especially well on clr-transformed data. Our COMBI method was best at identifying clusters of samples over the different views (see Figure 12). Our COMBI method and PCA with clr-transformation perform especially better in the ’no compensation’ scenario, indicating how crucial it is to account for compositionality. Detailed results can be found in Supplementary Section 5.

**Figure 10.**
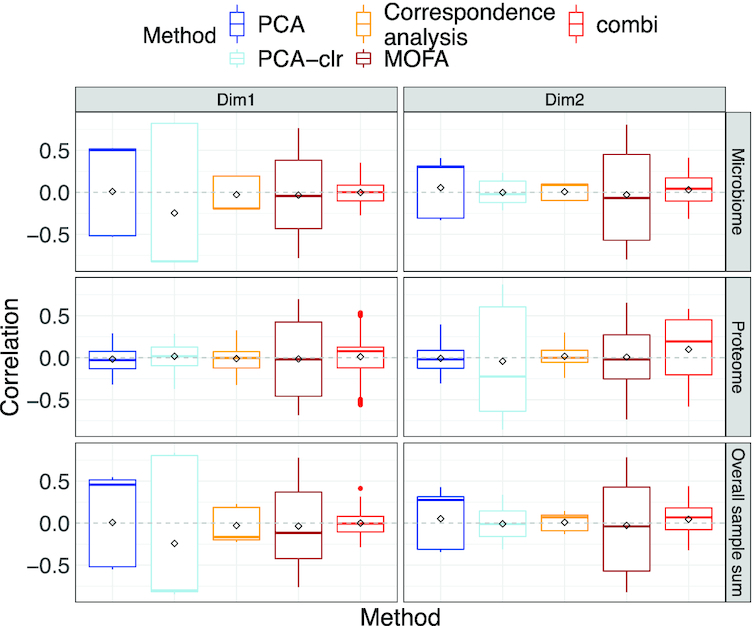
Simulation study. Boxplots of correlation between sample scores and overall and view-wise sample sums for permuted microbiome and proteome data simulated from the HMP2 dataset (strategy 3).

**Figure 11.**
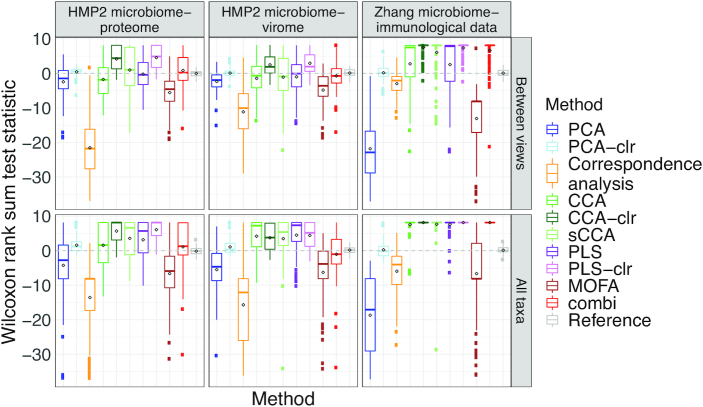
Simulation study. Wilcoxon rank sum test statistic quantifying correlated taxon identification for different methods (*x*-axis) and templates (top panels) on parametrically generated data based on the real case studies (strategy 1).

**Figure 12.**
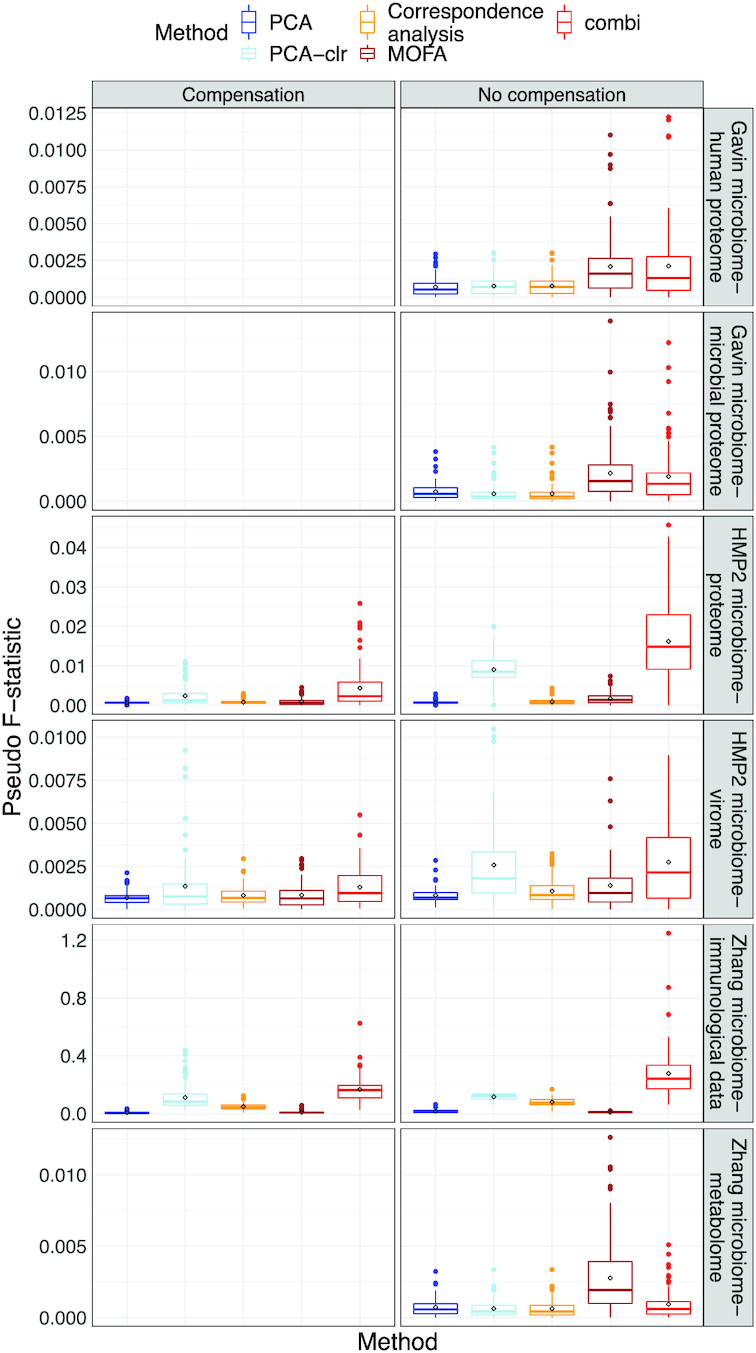
Simulation study. Boxplots of pseudo-F statistic (*y*-axis) quantifying sample separation for different methods (*x*-axis) and templates (top panels) under parametric simulation (Strategy 1).

## DISCUSSION

Data integration is challenging in statistical data analysis. Most statistical methods focus on a single dataset at a time, but integration requires finding common patterns across different views. Data integration can answer important biological questions, but sets a complicated task due to differences in measurement technology, outcome distributions and signal-to-noise levels. We have used dedicated regression models and outcome distributions for each view separately, whilst including common latent variables to discover signals across the different views. This results in a method enabling graphical exploration of multiple datasets. All features are included in the model, but visualization displays only features with the strongest signal. Thus, feature selection (e.g. by imposing sparsity) is deferred to later stages of the analysis with other methods. Alternatively, penalization could be included in a future version of *COMBI* for automated feature selection.

Another advantage of our method is its ability to handle missing data, that frequently arise in data integration problems—some views may not be measured on some samples. COMBI avoids the need of a complete case analysis or imputation thanks to explicit estimating equations. Thus, our method naturally ignores missing values in the fitting process without the need to drop samples. As with all other methods that account for missingness, this approach may still be biased when data are not missing at random. However, COMBI focuses on visualization rather than formal inference, and we believe this problem is less severe here in such framework. Nevertheless, all data analysts should always think carefully about the consequences of the study design and the causes of missingness.

In our method, we considered log-ratio link functions in regression models as a promising avenue for modelling compositional (count) data. Such models combine compositional effects with adequate mean-variance modelling and flexibility to include covariates. However we also face the same limitation as any classical compositional methods in terms of the interpretation of the results, which should be done with caution (27). Moreover, fitting these complex regression models on sparse omics datasets can be numerically challenging. Future investigations into their numerical properties, choice of link function and fitting algorithms are needed.

Finding an adequate distribution for sequence count data has proven difficult. Yet it is necessary to account for the unmistakable mean-variance trend of count data in ordination methods, to avoid undue interference of sequencing depths. We proposed a distribution-free approach for estimating the trend between relative abundance and variance by leveraging from the high dimensionality of the data. Contrary to most other data integration methods, our approach successfully avoids any influence of the sequencing depths on the samples scores.

Our COMBI method performed well at clustering samples with similar properties across different views, and is insensitive to differences in sequencing depth. Covariance-based methods such as canonical correspondence analysis and partial least squares are best at identifying correlated features, but do not yield overall sample ordinations, only distinct sample ordinations per view. Thus, the data analysts must choose their methods according to their research question and intended outputs. Our COMBI method offers an interesting alternative to existing data integration approaches when the focus is on exploring the relationship between samples and revealing which features contribute to differences between samples. Its constrained variant allows to include sample variables in the analysis, an appealing feature to deal with complex studies. As such, COMBI is a powerful new tool for the simultaneous exploration of multiple datasets.

## SOFTWARE

The R-package *combi*, which implements the integration algorithm, is available from BioConductor, with a detailed exemplar vignette.

## Supplementary Material

lqaa050_Supplemental_File
