# Supplementary material for: Model-based joint visualization of multiple compositional omics datasets
Source: NAR Genom Bioinform. 2020 Jul 21;2(3):lqaa050. doi: 10.1093/nargab/lqaa050 (PMC7671331; doi:10.1093/nargab/lqaa050)
Supplement: lqaa050_Supplemental_File [file lqaa050_supplemental_file.pdf]

Model-based joint visualization of multiple compositional omics datasets

Supplementary material

Stijn Hawinkel, Luc Bijnens, Kim-Anh Lê Cao and Olivier Thas

This document provides supplementary information to the article "Model-based joint visualization of multiple compositional omics datasets".

Contents

1 The data integration model **2**
1.1 Model specification . . . 2
1.1.1 Independence model . . . 2
1.1.2 Conditioning on baseline covariates . . . 2
1.1.3 Restrictions . . . 3
1.2 Model estimation . . . 3
1.2.1 Starting values . . . 4
1.2.2 Solving estimating equations for compositional data . . . 4
1.2.3 The abundance-variance trend . . . 4
1.2.4 Microarray data . . . 5
1.2.5 Latent variable estimation . . . 5
1.3 Influence measures . . . 6
1.4 Remarks about compositional data analysis . . . 6

2 Visualization **9**
2.1 Multiplots . . . 9
2.2 Compositional multiplots . . . 9
2.2.1 Intepretation with respect to all other features . . . 9
2.2.2 Interpretation with respect to other features in the same view . . . 10
2.2.3 Interpretation between features of different views . . . 11

3 Real data examples **12**
3.1 HMP2 data . . . 12
3.1.1 Microbiome-virome integration . . . 12
3.1.2 Microbiome-proteome-virome integration . . . 16
3.2 Zhang data . . . 18
3.2.1 Microbiome-immunological data integration . . . 18
3.2.2 Microbiome-metabolome integration . . . 21
3.3 Gavin data . . . 23

4 Methods comparisons **25**
4.1 Principal components analysis and correspondence analysis . . . 25
4.2 Canonical correlation analysis . . . 25
4.3 Partial least squares . . . 25
4.4 MOFA . . . 25

4.5 JIVE . . . 25

5 Simulation study **26**
5.1 Correlation of sample scores with library sizes . . . 26
5.2 Identification of correlated features . . . 35
5.3 Sample clustering . . . 36

6 Software **37**

1 The data integration model

1.1 Model specification

We specify the following statistical model:

$$\begin{aligned} g_x(E(\mathbf{X}|\mathbf{Z})) &= \mathbf{U}_x + \mathbf{Z}\mathbf{\Gamma} \\ g_y(E(\mathbf{Y}|\mathbf{Z})) &= \mathbf{U}_y + \mathbf{Z}\mathbf{\Theta} \end{aligned} \tag{1}$$

whereby g_x and g_y are appropriate link functions. $\mathbf{U}_x$ and $\mathbf{U}_y$ are offset matrices correcting for differences in baseline expression/abundance and sequencing depth defining the “independence model” (see next section). $\mathbf{Z}$ (n×M) is a low dimensional matrix of sample scores on latent variables (M = 2 or 3) [1]. $\mathbf{\Gamma}$ (M×p) and $\mathbf{\Theta}$ (M×q) are view-wise parameter matrices.

Note that model (1) is not a matrix decomposition of $\mathbf{X}$ and $\mathbf{Y}$. Instead it can be regarded as a low dimensional approximation of the expectation matrices of the saturated models E($\mathbf{X}$)=$\mathbf{X}$ and E($\mathbf{Y}$)=$\mathbf{Y}$, without calculating the entire decomposition.

1.1.1 Independence model

The first step of the fitting procedure is to estimate the independence models, i.e. the models describing homogeneous sample composition. These independence models defining the offset matrix are of the form

$$\begin{aligned} g_x(E_{indep}(\mathbf{X})) &= \mathbf{U}_x = \mathbf{d}_x\mathbf{e}_x^t \\ g_y(E_{indep}(\mathbf{Y})) &= \mathbf{U}_y = \mathbf{d}_y\mathbf{e}_y^t \end{aligned} \tag{2}$$

Whereby $\mathbf{d}_x$ en $\mathbf{d}_y$ are vectors of length n that quantify total sample abundance/expression, e.g. sequencing depth or array intensity. $\mathbf{e}_x$ and $\mathbf{e}_y$ are vectors of length p and q that quantify baseline feature means. They correct for baseline differences; independence models are *marginal* models. For compositional data, the restriction $\sum_{j=1}^{p} g_x^{-1}(e_{xj}) = 1$ is imposed.

1.1.2 Conditioning on baseline covariates

A next, optional step is to condition on known confounding variables such as batch or research center. Although simple in our regression framework, it is an important advantage over decomposition based methods. The confounding variables need not be identical for all views. We call the design matrices of two sets of (potentially overlapping) confounding variables $\mathbf{R}$ and $\mathbf{S}$, then the mean models can be extended such that:

$$\begin{aligned} g_x(E(\mathbf{X}|\mathbf{R})) &= \mathbf{U}_x + \mathbf{R}\mathbf{\Phi} \\ g_y(E(\mathbf{Y}|\mathbf{S})) &= \mathbf{U}_y + \mathbf{S}\mathbf{\Xi} \end{aligned} \tag{3}$$

In case of compositional data, one restriction is needed to guarantee a solution. In this case we use the additive log-ratio (alr) transform [2] as link function, which is defined as:

$$alr(\mathbf{x}) = \log\Big(1, \frac{x_2}{x_1}, \frac{x_3}{x_1}, \frac{x_4}{x_1}, ..., \frac{x_p}{x_1}\Big). \tag{4}$$

This effectively sets the parameter of the first feature of a view to zero for all confounders ($\boldsymbol{\phi}_{.1} = \mathbf{0}$).

Another problem occurs for discrete confounders with features that only have zero observations in one of the groups defined by these confounders. One could filter out all these features, but this leads to significant data loss. An alternative solution is offered by bias-reduced estimates [3, 4, 5]. Instead of correcting the bias of the maximum likelihood estimates (which are infinite under the scenario above), they reduce the bias by directly modifying the estimating equations. This allows for the estimation of the confounder parameters under this scenario. If the (quasi)-score equation for a mean parameter η is given by $\mathbf{s}_\eta$, then the bias-reduced (quasi)-score equation is of the form

$$\mathbf{s}_\eta + \mathbf{A}_\eta = \mathbf{0}$$

with $\mathbf{A}_\eta = R^t\xi$. Hereby $\xi_i = \frac{h_i}{(2f_i)}f_i'$ with h_i the diagonal elements of the *hat matrix* $\mathbf{R}(\mathbf{R}^t\mathbf{R})^{-1}\mathbf{R}^t$ and $f_i = \frac{d\mu_i}{dg_x(\mu_i)}$ and $f_i' = \frac{d^2\mu_i}{dg_x(\mu_i)^2}$. Hence $f_i = \big(\frac{d\log(\mu_i)}{d\mu_i}\big)^{-1} = \mu_i$ and $f_i' = \mu_i$ such that $\xi_i = \frac{-h_i}{2}$. In practice, these systems of estimating equations are still very hard to solve though, because of the near singularity of the Jacobian matrices.

1.1.3 Restrictions

Model (1) is overspecified, as both the latent variables and coefficients are unknown and need to be estimated from the data. Hence restrictions need to be applied to guarantee an identifiable model. The columns of $\mathbf{Z}$ are restricted to be orthogonal: $\mathbf{Z}^T\mathbf{Z} = \text{diag}(\boldsymbol{\psi})$ with *diag()* defining a diagonal matrix with the vector $\boldsymbol{\psi}$ with non-negative entries on the diagonal. The coefficient matrices are restricted to be orthonormal: $\boldsymbol{\Gamma}\boldsymbol{\Omega}_x\boldsymbol{\Gamma}^T$ $= \boldsymbol{\Theta}\boldsymbol{\Omega}_y\boldsymbol{\Theta}^T = \mathrm{I}_M$, with $\boldsymbol{\Omega}_x$ and $\boldsymbol{\Omega}_y$ view specific, diagonal weight matrices and I_M the identity matrix of dimension M. The choice of these weights follows Hawinkel et al. [6]: all samples are considered equally likely to be drawn from the population and receive equal weights in the restrictions. For the features, more abundant features are considered to be more likely to be drawn from the population and receive weights (on the diagonal of $\boldsymbol{\Omega}$) proportional to their abundance under the independence model $g^{-1}(\mathbf{e})$.

Note that because the model is overspecified, classical inference based on (quasi-)likelihood does not hold. No confidence intervals or p-values can be calculated. The model is intended for data exploration only and relies solely on the point estimates.

Technical note: Centering and orthogonalization restrictions are directly imposed in the optimization procedure through Lagrange multipliers. This is crucial to avoid overflow, i.e. certain numbers becoming to large for the computer to store. Normalization restrictions can be imposed *post hoc*. This does not cause numerical problems, and the iterative algorithm will not stop until they are fulfilled. This approach is faster, as the initial optimization problem is simpler. Even if the estimating equations were not solved in some iteration, afterwards the centering and orthogonalization will be still be enforced through Gram-Schmidt orthogonalization to speed up convergence. Finally, some restriction is needed to render the estimation under compositionality with centered log-ratio transform feasible, but the centering restrictions already conveniently fulfill this role.

1.2 Model estimation

In summary, the fitting algorithm consists of the following steps

1. Estimate the view-wise independence models by iterating between the estimation of the row and column offsets and possible nuisance parameters (e.g. standard deviations for microarray data, abundance-variance trends for sequence count data).
2. (Optional) Estimate feature parameters for confounding variables, and condition on them by including their contribution to the mean matrix in the offset. This step also occurs independently for each view.
3. Iterate between estimating the latent variables, feature parameters and possible nuisance parameters. When convergence for one dimension is achieved, incorporate this dimension in the offset, and estimate the next dimension conditional on all previous ones.

The iterative procedures in steps 1 and 3 continue until convergence. Convergence is declared when the square root of the L_2 norm of the all estimates drops below a certain tolerance (here 1e-4), e.g. for the latent variables:

$$\sqrt{\left(\frac{\mathbf{Z}^{old}_{.m} - \mathbf{Z}^{new}_{.m}}{\mathbf{Z}^{old}_{.m}}\right)^t \left(\frac{\mathbf{Z}^{old}_{.m} - \mathbf{Z}^{new}_{.m}}{\mathbf{Z}^{old}_{.m}}\right)} < 10^{-4},$$

with $\mathbf{Z}^{new}_{.m}$ the current estimates and $\mathbf{Z}^{old}_{.m}$ the estimates of the previous iteration. It may be prudent to graphically check for convergence. An example of such convergence plot is shown in Figure S16.

1.2.1 Starting values

Iterative algorithms converge much faster when provided with reasonable starting values. For the independence model, simple row and column sums can be used. Starting values for latent variables and feature coefficients can be obtained from following singular value decompositions. With offset matrices $g_x^{-1}(\mathbf{U}_x)$ and $g_y^{-1}(\mathbf{U}_y)$, obtain standardized residual matrices $\frac{\mathbf{X}-g_x^{-1}(\mathbf{U}_x)}{\mathbf{s}v_{indep}(\text{clr}^{-1}(\mathbf{e}^t))}$ (for sequence count data) or $\frac{\mathbf{Y}-g_y^{-1}(\mathbf{U}_y)}{\text{diag}(\sigma_{indep})}$ (for microarray data). σ_{indep} are the column wise standard deviations under the independence model); see Section 1.2.3 for the denominator of the sequence count case. These residual matrices are concatenated by row into one large matrix $\mathbf{D}$, for which the singular value decomposition is then obtained:

$$\mathbf{D} = \mathbf{G}\boldsymbol{\Sigma}\mathbf{H}^t$$

The first M columns of $\mathbf{G}\boldsymbol{\Sigma}$ are then used as starting values for $\mathbf{Z}$, and the first M columns of $\mathbf{H}$ as starting values for the corresponding feature parameters. For a constrained analysis, redundancy analysis [7] on the matrix $\mathbf{D}$ and the design matrix of baseline sample variables $\mathbf{c}$ is used to obtain starting values for the environmental gradients and feature parameters.

1.2.2 Solving estimating equations for compositional data

The Newton-Raphson algorithm that is used to solve the estimating equations may encounter local extrema and saddle points when applied to compositional data. Therefore, if the Newton-Raphson algorithm does not converge when the old parameter estimates are used as stating values, random standard normal variates are repeatedly used as starting values instead until the estimation equations are solved. This is a brute-force solution but it works in most cases. This kind of problems in high dimensional estimation problems are very intractable, more theoretical research is needed into a more general solution in the compositional framework.

1.2.3 The abundance-variance trend

The abundance-variance trend models the relationship between $\log(\pi_j)$ and $\log\left(\overline{\text{Var}(X_j|\mathbf{Z}_{.1,m})}\right) = \log\left(\frac{1}{n-1}\sum_{i=1}^n \frac{(X_{ij}-E(X_{ij}|\mathbf{Z}_{.1,m}))^2}{s_i}\right)$ through a smooth function a_m. The fit is performed on the log-scale since this spreads the observations nicely and avoids undue influence of extreme values. It allows to predict

$\text{Var}(X_{ij}|\mathbf{Z}_{.1,m})$ as $v_m(\pi_{ij})s_i = \exp\big(a_m[\log(\pi_j)]\big)s_i$. This approach shares information between features to reliably estimate variances. The smooth function a_m is updated throughout the iterative procedure and is unique to every dimension. Note that this approach may imply that for some observations X_{ij} and $X_{i'j'}$, $\exp\big(a_m(\log(\pi_j))\big)s_i \neq \exp\big(a_m(\log(\pi_{j'}))\big)s_{i'}$ even though $E(X_{ij}|\mathbf{Z}_{.1,m}) = E(X_{i'j'}|\mathbf{Z}_{.1,m})$.

Anders et al. [8] use a local regression as a smooth function, but this may yield a smoother with a irregular derivatives. This destabilizes the Newton-Raphson algorithm used to solve the estimating equations. Instead we use a natural smoothing spline, which has continuous first and second order derivatives. At the cost of being slightly more rigid, it yields a much more stable algorithm.

Great care should be taken when extrapolating this abundance-variance trend to small relative abundances. This will be necessary as the modelling processes may yield small relative abundances for some features in some samples. It is known that for small means, sequence count data approximately follow the Poisson distribution [9, 10]. The Poisson distribution has a variance that is equal to the mean. Hence, as a heuristic, for small abundances it is assumed that $\text{Var}(X_{ij}|\mathbf{Z}_{.1,m}) = \pi_j s_i$. The smooth function is then constrained to have slope 1 and equal the diagonal line for some value between $(\min_{\boldsymbol{\pi}}\{\log(\pi_j)\} - 1)$ and $\log\Big[\Big(\sum_{i=1}^n \sum_{j=1}^p E(X_{ij}|\mathbf{Z}_{.1,m})\Big)^{-1}\Big] - 10$. This value is selected as the one that minimizes the squared error

$$\sum_{j=1}^{p}\Big(\text{Var}(X_{ij}|\pi_j, s_i) - \exp(a(\log(\pi_j)))s_i\Big)^2.$$

This complicated solution is motivated by the need to keep the derivatives continuous for numerical stability.

1.2.4 Microarray data

For modelling microarray data, we mainly follow the tracks of the popular *limma* package [11]. The array data is log-transformed, and then modelled using a simple linear model with identity link. The estimates of the feature-wise variances are shrunken towards a common value using an empirical Bayes procedure [12]. The estimating equations are then:

$$\sum_{i=1}^{n} Z_i \frac{Y_{ij} - \mu_{ij}}{\sigma^2_{j,EB}}$$

with $\mathbf{Y}$ the microarray data matrix. $\sigma^2_{j,EB}$ is the empirical Bayes estimate of the variance for feature j.

1.2.5 Latent variable estimation

The estimating equations for the latent variables are obtained by summing the estimating equations of all different views for every sample. If desired, different weights can be allotted to the different datasets in this way, but we use even weights by default. If all weight is allotted to a single dataset this reduces to a single view problem, as e.g. the *RCM* package [6].

One reasoning is to inverse weigh the elements of the estimating equations for the latent variables by the number of features in the view. Otherwise views with many features might get a very strong impact on the estimation of the latent variables, without there being a biological rationale why they should contain more information. Another argument is to state that datasets with more features carry more information and can have more weight in the estimation. Finally, it may be that the dataset with the clearest signal will take preponderance. An answer to these questions is given below in section 1.3.

1.3 Influence measures

The impact of each of the views on the estimation on the latent variables or environmental gradient components can be obtained through influence functions [13]. Influence functions reflect the influence a certain observation has on a parameter estimate, keeping the other sorts of parameters fixed. Because of the iterative algorithm this latter assumption is incorrect, but the influence functions may still harbour interesting information.

For maximum likelihood estimation, the influence function $\chi(\eta|f, \mathbf{x})$ of a parameter η for a distribution f and data $\mathbf{x}$ is defined as:

$$\chi(\eta|f, \mathbf{x}) = -\mathbf{S}_f(\eta|\mathbf{x})E\big[\mathbf{I}(\eta|f)\big]^{-1}$$

with $\mathbf{S}_f(\eta|\mathbf{x})$ the score function and $E\big(\mathbf{I}(\eta|\mathbf{x})\big)$ the expected Fisher information matrix. We use the same concept for the quasi likelihood estimation, by replacing $\mathbf{S}_f(\eta|\mathbf{x})$ by the quasi score functions and $E\big(\mathbf{I}(\eta|f)\big)^{-1}$ by the Jacobian matrix. If an observation has a positive influence on a parameter, it means that it tries to "pull its value up". In other words, if the observation would not be there, the parameter estimate would be lower. As the orientation of the final graph is of no importance, it is often sufficient to look at absolute influences.

As an illustration, a dataset with three views was generated using the negative binomial distribution. The number of features are 100, 100 and 1000 respectively. The signal strength (i.e. the fold changes) is the same in all datasets. The first and third datasets have similar levels of overdispersion, the second dataset has high levels of overdispersion. We call these datasets the "regular", "noisy" and "large" datasets. In Figures S2-S4 it is demonstrated that signal-to-noise ratio of each view drives its influence, rather than the number of features. The noisy dataset has least influence, whereas the influence of the regular and large datasets is comparable.

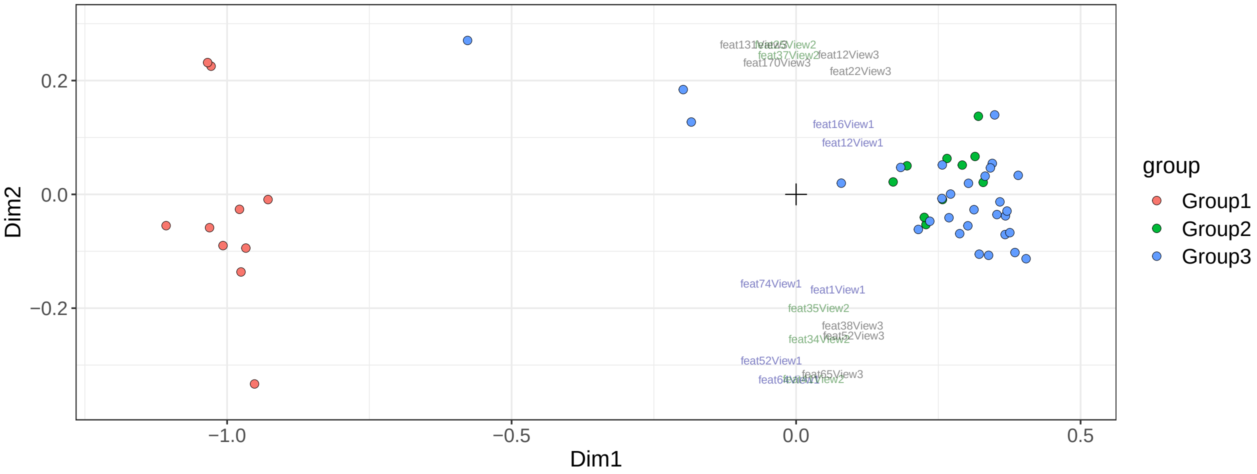

Figure S1: Data integration multiplot of synthetic dataset for the illustration of influence measures.

1.4 Remarks about compositional data analysis

The use of the centered log-ratio transform does not guarantee subcompositional coherence for the integration model. Subcompositional coherence means that the conclusions for features j and j' do not change when a third feature j'' is omitted from the analysis (e.g. filtered out) [14]. However, when a taxon is omitted, the geometric mean of the composition changes, and thus also the outcome of the ordination. Also, because of the iterative nature of the procedure, omitting taxon j'' will change the estimates of the latent variables. This will in turn change the estimates of the feature parameters j and j', so that the procedure is not

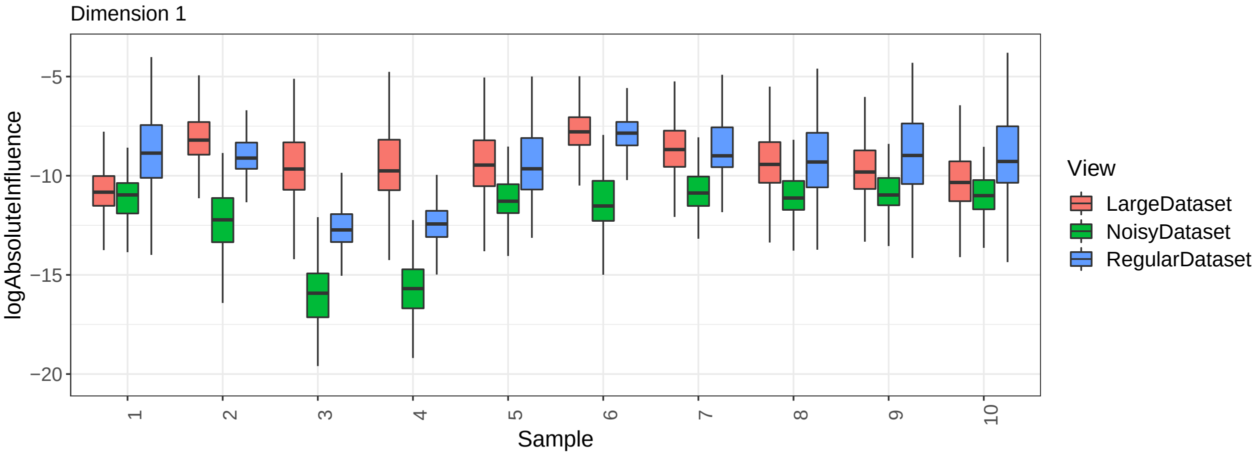

Figure S2: Total influence of each view on the estimation of the latent variables in dimension 1 for the synthetic dataset of Figure S1. Values of latent variables are shown as black crosses. The noisy dataset has least influence over the estimation of the latent variables. The large and regular datasets have similar influence, since they contain a similar signal and the same level of noise.

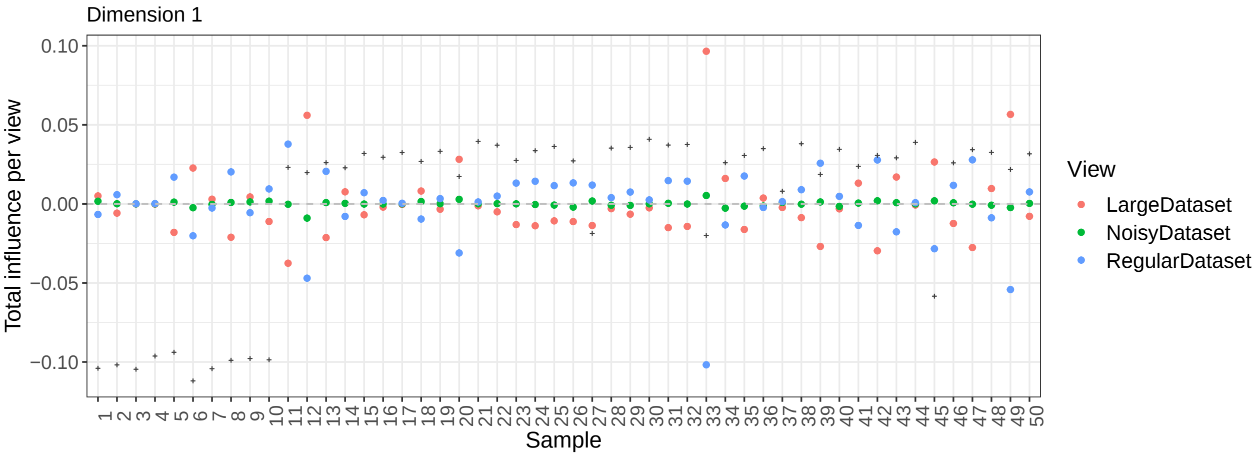

Figure S3: Point plot of absolute values of influences of the feature on the estimation of the latent variables, per view. As expected, average influences are zero (since the algorithm has converged). The regular and large dataset have similar influence.

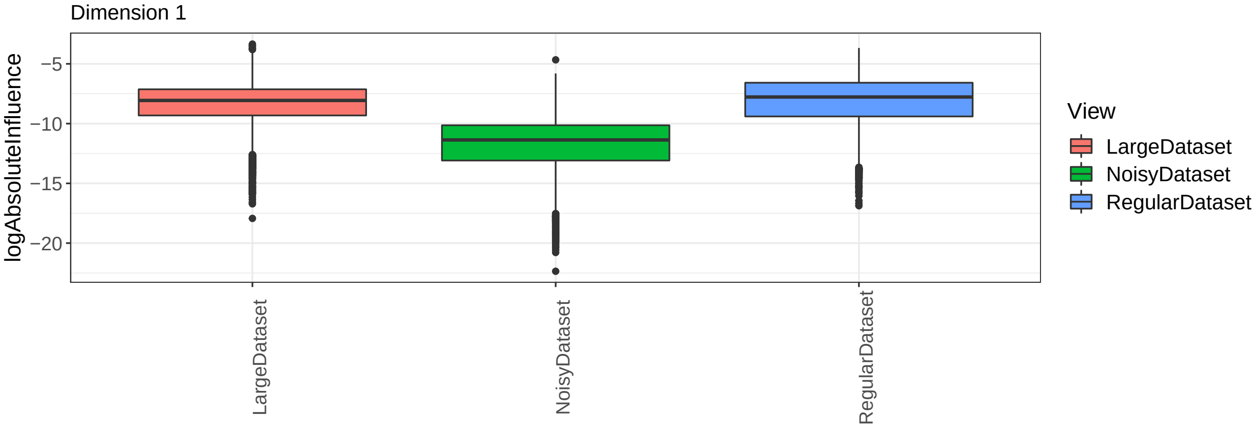

Figure S4: Boxplots of overall absolute influences of the views on the estimation of the latent variables for the synthetic dataset of Figure S1. It is clear that the features from the noisy dataset have less influence.

subcompositionally coherent. Because of the same reasons, classical CoDa biplots of log-ratio transformed data are also not subcompositional coherent.

Neither is our method scale invariant as is sometimes stated as a requirement for compositional data analysis [14, 15]. Scale invariance means that the analysis should not depend on the total size if the composition, e.g. the library sizes in case of sequence count data. While it is true that the conclusions are only drawn on the proportions, the sampling variability of the proportions depends on the library size. A sample with 1.000.000 reads carries much more information than a sample with only 10.000 reads, even when the compositions are identical. Hence analysis of heteroscedastic count data should never be fully scale invariant. Ignoring the mean-variance trend in sequence count data leads to technical artefacts in CoDa biplots (see Hawinkel et al. [16], Supplementary material Section 3.4.1), as is also demonstrated below in Section 5.1.

2 Visualization

2.1 Multiplots

The resulting data integration can be visualized in a *multiplot* as follows:

1) Build an orthogonal axis system with equally scaled axes in 2 (or 3) dimensions. Plot the first dimensions of $\mathbf{Z}$ as dots. As the dimensions of $\mathbf{Z}$ are orthogonal, but not normalized, the distances between the samples reflect the dissimilarities between the samples over all different views.
2) Add labels at the locations defined by $\mathbf{\Gamma}$ and $\mathbf{\Theta}$, with arbitrary scaling. As the components are orthogonal, the *biplot principle* holds [17], and the orthogonal projection of e.g. the vector from the origin to $\boldsymbol{\gamma}_j$ onto the vector from the origin to $\mathbf{Z}_i$ is proportional to the departure from independence of feature j in sample i for the dimensions plotted. Moreover, when the projection $\boldsymbol{\gamma}_j^t\boldsymbol{\gamma}_l$ is large, this indicates that features j from view $\mathbf{X}$ and l from view $\mathbf{Y}$ are similarly associated to latent variables $\mathbf{Z}$ and are thus correlated. As the feature parameters are also normalized, distances between feature parameters locations cannot be interpreted (it is a so-called *form* or *sample* multiplot). To avoid overplotting, it may be necessary to limit the features plotted to the ones with the largest norms (furthest away from the origin), i.e. thresholding.
3) In case of constrained ordination, the components of $\mathbf{\Lambda}$ can be added to the plot, as arrows or as labels, again with arbitrary scaling. The projection of these variable vectors $\boldsymbol{\lambda}_k$ onto $\boldsymbol{\gamma}_j$ reveals how sensitive feature j is to changes in variable k. Also, the larger the component $\boldsymbol{\lambda}_k$, the more important the variable k is in driving the variability over the different views.

The scaling in steps 2 and 3 is usually done such that all coordinates have the same order of magnitude as the sample location, to aid interpretability. Only the relative length of the projections is meaningful.

When some of the views consist of compositional data, the interpretation of the plot is complicated. For compositional data, a positive feature parameter $\boldsymbol{\gamma}_{mj}$ does not guarantee that the feature j is positively associated with the latent variable of dimension m. Neither does $\text{clr}^{-1}(\boldsymbol{\gamma}_m)_j > 1/p$ guarantee this, as wrongly suggested by Xia et al. [18]. The impact of the feature parameter on the mean of a feature j depends on the values of the other feature parameters of that view, as well as on the value of the latent variable. In an extreme case, for $z_{im} \to \infty$, the composition collapses into a point mass of 1 at taxon j with the highest γ_{mj}.

Once the model is fitted, the values of the latent variables and feature parameters are known of course. One can thus simply check that for those features with the largest loadings that would be plotted, the expected abundance does in fact vary monotonically with the latent variable within the observed range of latent variable values. Unfortunately, in practice this is almost never the case for most features. An explanation on how to interpret these biplots under this curse of compositionality is given below.

2.2 Compositional multiplots

Compositional biplots have been introduced by Aitchison et al. [19] for log-transformed data. The interpretation is the same though in our case of inverse log-transformed parameters, and is less intuitive than for a regular biplot. In a regular biplot, each combination of sample and feature labels is interpretable. In a compositional setting, a feature label can never be interpreted by itself, but should always be interpreted *relatively* to some other features. Here we discuss the interpretation with respect to 1) all other features of the same view, 2) one other feature of the same view and 3) a feature from another view.

2.2.1 Intepretation with respect to all other features

The interpretation with respect to all other features is the comparison with the geometric mean (gm) of all proportions:

$$\mathrm{gm}(\boldsymbol{\pi}) = \exp\big(1/p\sum_{j=1}^{p}\log(\pi_j)\big)$$

The gm behaves similarly to the Shannon index [20] in the sense that it can be seen as a measure of evenness. For a perfectly even species composition ($\pi_1 = \pi_2 = \ldots = \pi_p = 1/p$), the gm equals 1/p. As one feature becomes more and more abundant (one $\pi_j \rightarrow 1$), the gm approaches 0.

In our model, the log ratio of the proportion of one feature a on the gm of the proportions in sample i is a linear function of the latent variables:

$$\log\left(\frac{\pi_{ia}}{\mathrm{gm}(\boldsymbol{\pi}_i)}\right) = e_a + \mathbf{Z}_i^t\boldsymbol{\gamma}_a$$

The biplot can be interpreted as a regular biplot in function of this log-ratio: the larger the projection $\mathbf{Z}_i^t\boldsymbol{\gamma}_a$ becomes, the more this log-ratio departs from the independence model for feature a in sample i. Loosely speaking, the larger $\mathbf{Z}_i^t\boldsymbol{\gamma}_a$ becomes, the more *dominant* feature a becomes. How the proportion π_{ia} evolves as a function of $\mathbf{Z}_i$ depends on the numerical values of $\mathbf{e}$ as well as $\boldsymbol{\gamma}$. We can make this clear as follows (dropping sample subscripts, and looking at one dimension), knowing that:

$$\pi_a(Z) = \frac{\exp(e_a + Z\gamma_a)}{\sum_{j=1}^{p}\exp(e_j + Z\gamma_j)}.$$

Hence, taking the logarithm

$$\log\big[\pi_a(Z)\big] = e_a + Z\gamma_a - \log\big(\sum_{j=1}^{p}\exp(e_j + Z\gamma_j)\big)$$

This takes the shape of the regular log-linear model. To know how this proportion evolves with the latent variable, we take the derivative with respect to Z:

$$\frac{\partial\log\big[\pi_a(Z)\big]}{\partial Z} = \gamma_a - \sum_{j=1}^{p}\gamma_j\pi_j(Z)$$

The orthonormality restriction guarantees that $\sum_{j=1}^{p}\gamma_j\big[\mathrm{clr}^{-1}(\mathbf{e})\big]_j = \sum_{j=1}^{p}\gamma_j\pi_j^{indep} = 0$ for every dimension (see section 1.1.3). Hence we can also write

$$\frac{\partial\log\big[\pi_a(Z)\big]}{\partial Z} = \gamma_a - \sum_{j=1}^{p}\gamma_j\big(\pi_j(Z) - \pi_j^{indep}\big)$$

For small departures from independence the second term drops, but for realistic datasets this is not the case. In practice, the second term can even be larger than γ_a in absolute value, upsetting the monotonicity of π_a with γ_a. This formula cannot easily be simplified further, the interpretation will have to account for the compositionality. This potential pitfall in interpreting centered log-ratios is illustrated in Figure 2 in the main text.

2.2.2 Interpretation with respect to other features in the same view

As the interpretation with respect to "the rest of the features" (represented by the geometric mean) is so problematic, it may be easier to compare just two features. We look at the log-ratio between the relative abundances of two features π_a and π_b in sample i. According to the model:

$$\log\left(\frac{\pi_{ia}}{\pi_{ib}}\right) - \log\left(\frac{\pi_a^{indep}}{\pi_b^{indep}}\right) = \mathbf{Z}_i^t(\boldsymbol{\gamma}_a - \boldsymbol{\gamma}_b),$$

with $\pi_j^{indep} = \text{clr}^{-1}(\mathbf{e})_j$ the proportion of feature j under the independence model. Note that we have eliminated $\text{gm}(\boldsymbol{\pi})$ from the expression. The expression on the left hand side has the form of a log odds ratio as in logistic regression, but with the difference that $\frac{\pi_{ia}}{\pi_{ib}}$ and $\frac{\pi_a^{indep}}{\pi_b^{indep}}$ are not genuine odds.

The difference $(\boldsymbol{\gamma}_a - \boldsymbol{\gamma}_b)$ between vectors is known as the *link* in a plot, i.e. the straight line connecting the points defined by $\boldsymbol{\gamma}_a$ and $\boldsymbol{\gamma}_b$. It is small when the labels $\boldsymbol{\gamma}_a$ and $\boldsymbol{\gamma}_b$ lie on approximately the same side of the origin and at the same distance from it ($\boldsymbol{\gamma}_a \approx \boldsymbol{\gamma}_b$). In that case the ratio of the relative abundances $\frac{\pi_{ia}}{\pi_{ib}}$ will not differ from that under the independence model $\frac{\pi_a^{indep}}{\pi_b^{indep}}$ by much in *any* sample. In a compositional setting, a stable ratio means that the features are strongly correlated [14].

In case this link is large, the projection of the latent variable vector $\mathbf{Z}_{i.}$ onto the link (i.e. $\mathbf{Z}_i^t(\boldsymbol{\gamma}_a - \boldsymbol{\gamma}_b)$) indicates how much and in which direction the ratio $\frac{\pi_{ia}}{\pi_{ib}}$ differs from that under the independence model [19]. Note that this implies that feature labels lying at the same side of the origin but at different distance (i.e. $\frac{\boldsymbol{\gamma}_a}{||\boldsymbol{\gamma}_a||} = \frac{\boldsymbol{\gamma}_b}{||\boldsymbol{\gamma}_b||}$ but $||\boldsymbol{\gamma}_a|| \neq ||\boldsymbol{\gamma}_b||$) are not necessarily strongly correlated in all samples! The interpretations discussed above are illustrated graphically in Figures 3 and S7-S8.

2.2.3 Interpretation between features of different views

The interpretation between features of different, compositional views, or between features from a compositional view and a non-compositional view is even more difficult. Of course the interpretation with respect to the centered log-ratio is always valid, but not intuitive. If labels of feature a in view 1, and feature b in view 2 lie at the same side of the origin, their centered log-ratio transforms are correlated. This means that moving along this direction, both features become “more dominant” in their own views, although this need not imply that their abundances also increase. Features from two non-compositional views are correlated if they lie on the same side of the origin.

3 Real data examples

In this section the data integration plots of real data are shown for the integrations that were not shown in the main paper.

3.1 HMP2 data

The Human Microbiome Project 2 (HMP2), or integrative HMP (iHMP), aims to investigate the relationship between the microbiome and host responses. It extends the original, cross sectional HMP by also including longitudinal samples. Here we focus on the datasets in the "The Inflammatory Bowel Disease Multi'omics Database" (IBDMDB), which contains healthy and IBD patients (patients with both forms of IBD, Crohn's disease (CD) and ulcerative colitis (UC), are included), see the project website. A total of 90 subjects was be profiled for one year. The HMP2 dataset contains many different types of omics data, from which we selected the following.

The microbiome composition of the stool was assessed through sequencing of the 16S rRNA gene. The proteome was measured in fecal, nasal and blood samples. Proteins were separated by liquid chromatography and then identified using mass spectroscopy. This yields counts of proteins. The proteins were then classified biochemically (EC) or phylogenetically (KO). We use the latter convention here. Proteomics data are also to be considered compositional [21]. The composition of the virome of the stool was measured by sequencing marker genes as for the microbiome data. Data integration multiplots of these datasets can be found in Figures S5-S10.

3.1.1 Microbiome-virome integration

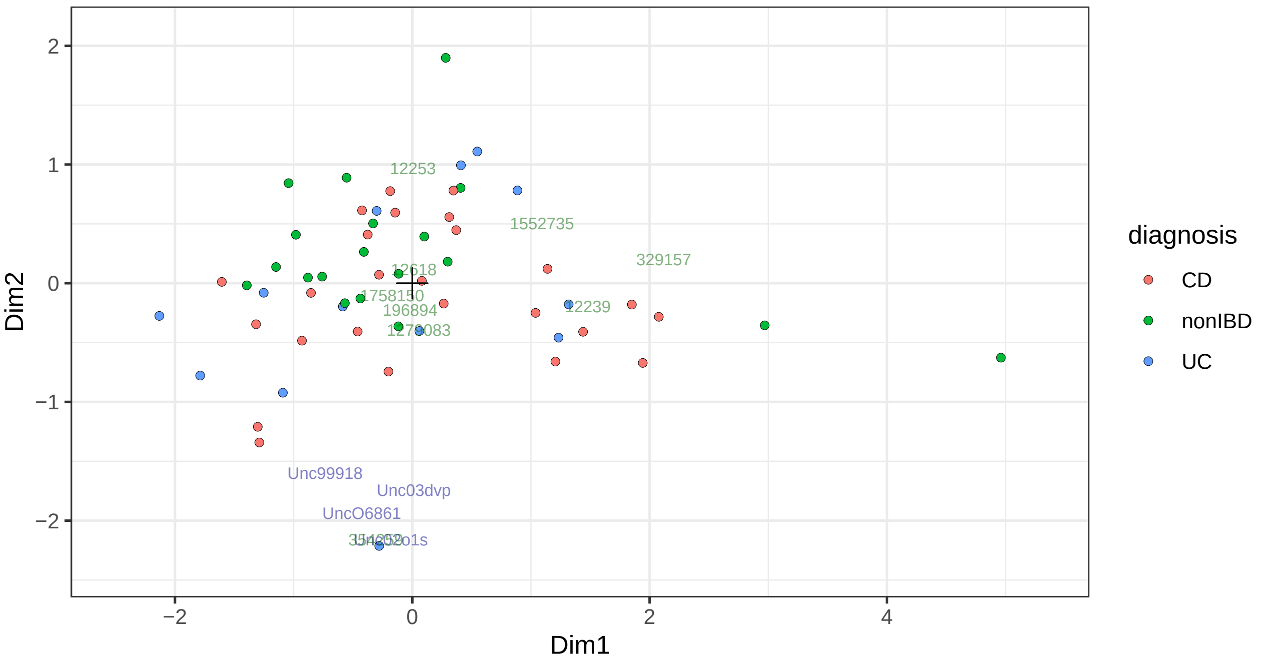

Figure S5: Data integration plot of microbiome and virome data from the HMP2 project. Viral taxa are shown in green, bacterial taxa in blue

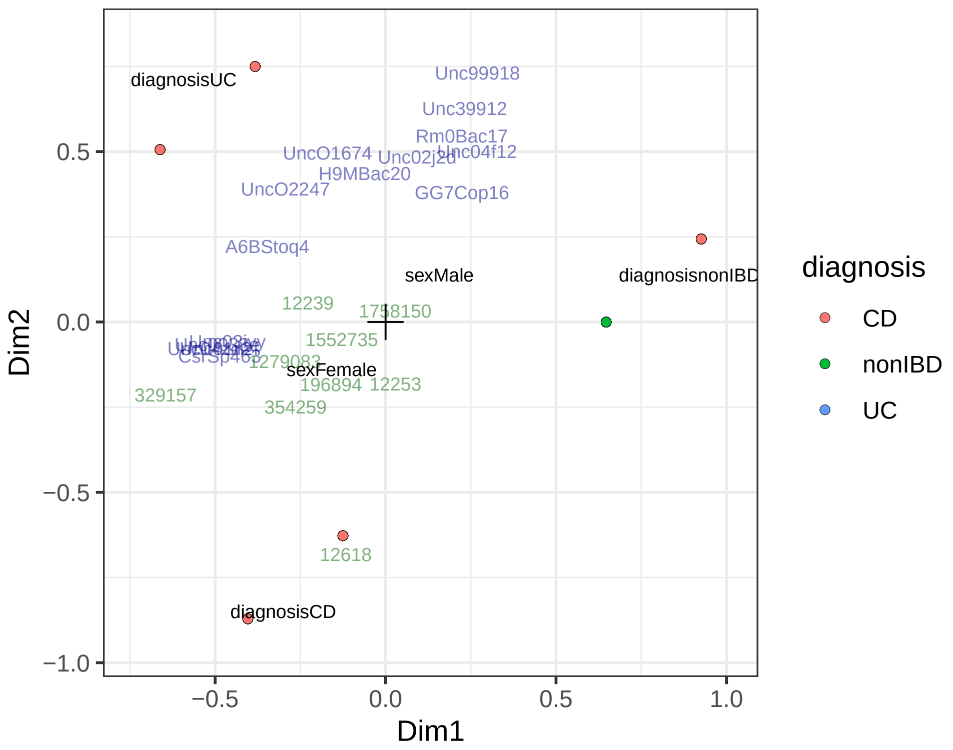

Figure S6: Constrained ordination of HMP2 microbiome and virome data. Viral taxa are shown in green, bacterial taxa in blue, patient variables in black.

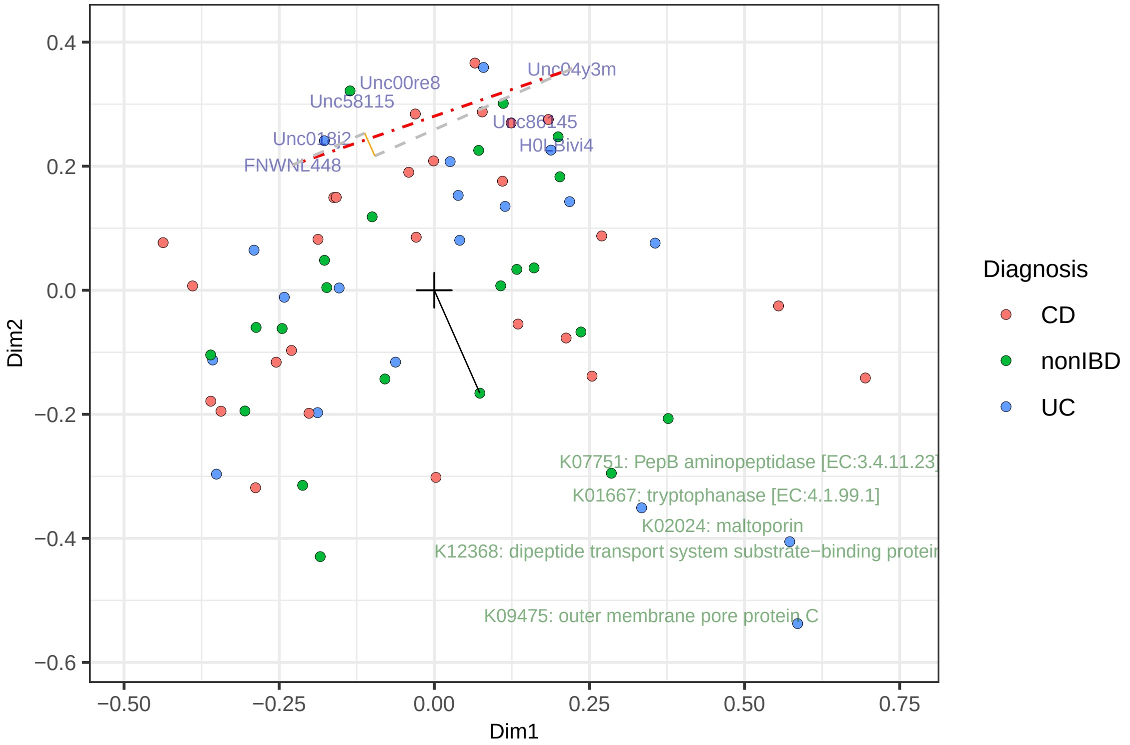

Figure S7: Data integration plot of microbiome and proteome data from the HMP2 project. Coloured dots represent patients, labels represent features of microbiome (blue) and proteome (green). The red dashed line shows the link between taxa *Unc04y3m* and *FNWNL488*, the orange line its projection onto the non-IBD sample vector bottom right. This projection is small, such that the ratio FNWNL448/Unc04y3m is not different in this sample from the average sample.

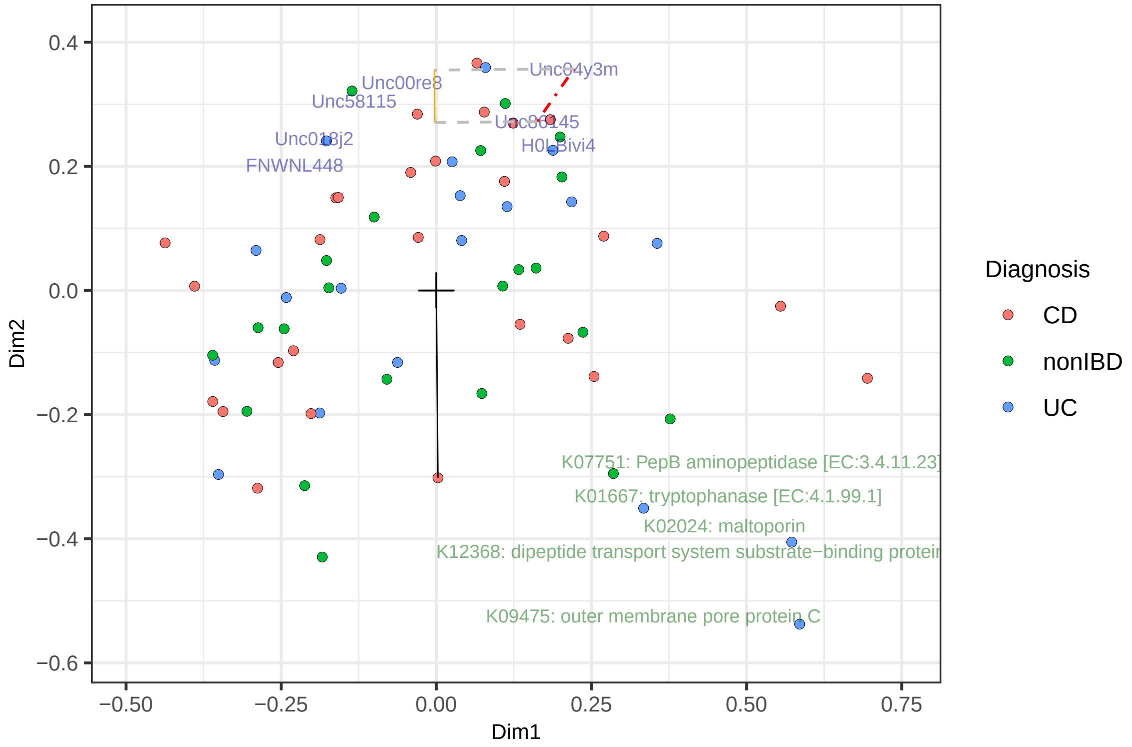

Figure S8: Data integration plot of microbiome and proteome data from the HMP2 project. Coloured dots represent patients, labels represent features of microbiome (blue) and proteome (green). The red dashed line shows the link between taxa *Unc86145* and *Unc04y3m*, the orange line its projection onto the CD sample vector on the bottom. Despite the two taxa lying at the same side of the origin, the projection onto the sample vector is not zero, and hence ratio Unc86145/Unc04y3m is larger in this sample than in the average sample.

3.1.2 Microbiome-proteome-virome integration

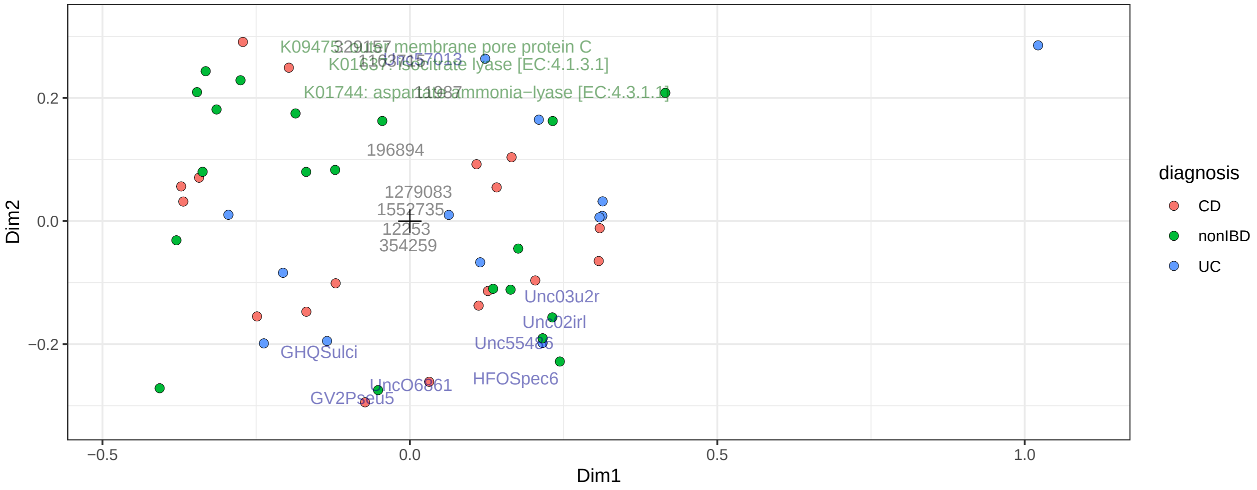

Figure S9: Quadriplot of HMP2 microbiome, proteome and virome data integration. Corresponding features are represented in blue, green and red.

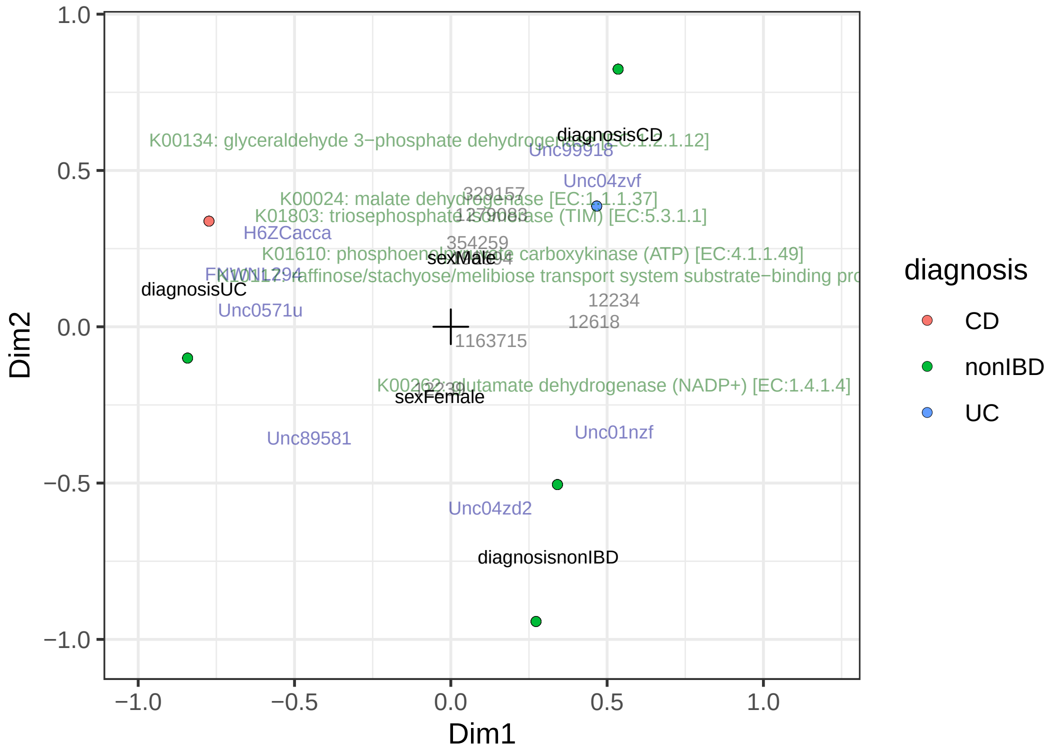

Figure S10: Pentaplot of HMP2 microbiome, proteome and virome constrained data integration. Corresponding features are represented in blue, green and red.

3.2 Zhang data

This study investigated the effect of one or three pulsed antibiotic treatments (1 and 3 PAT) on the onset of type I diabetes in mice [22]. Many views were measured, including gut microbiome, metagenomics, metabolic pathways and intestinal immunity pathways. Microbiome composition determined through 16S sequencing. Intestinal immunity pathways measured using Nanostring. This is basically expression profiling but focused on a subset of genes involved in immunity. The original publication focused on the effect of the PAT on all different views, without attempting to integrate the different views.

3.2.1 Microbiome-immunological data integration

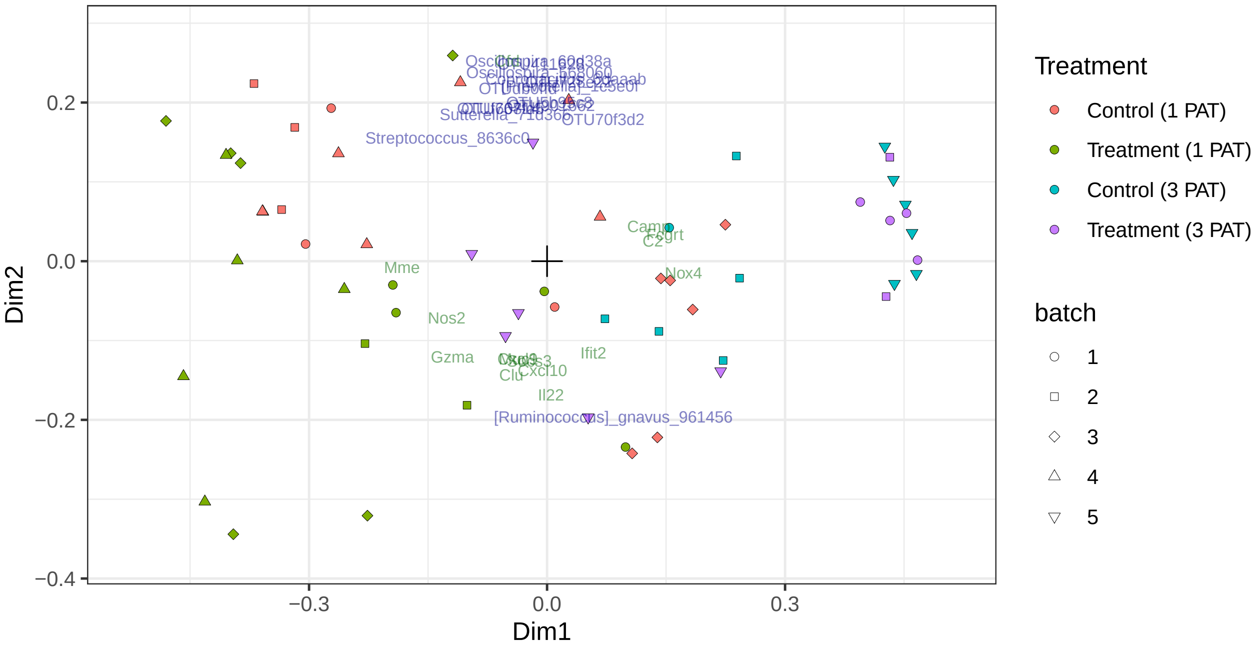

Figure S11: Data integration of microbiome and immunological Zhang data. The respective features are shown as blue and green labels.

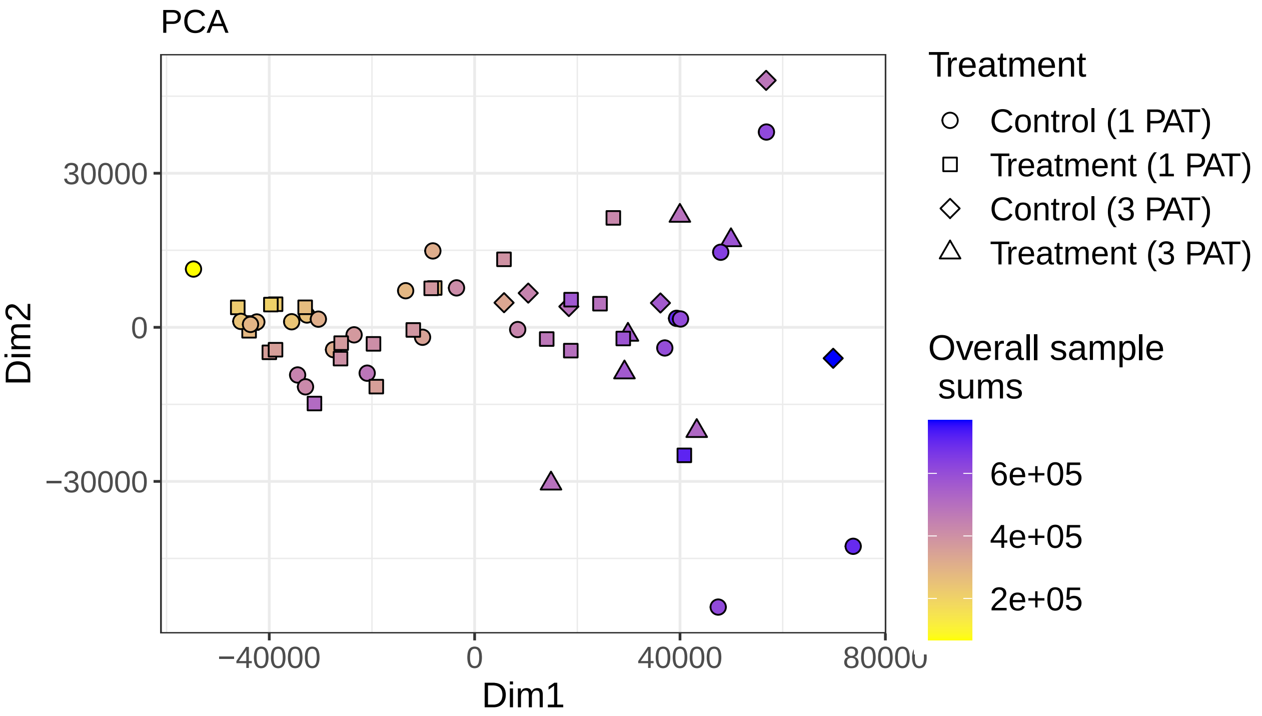

Figure S12: Sample ordination of Zhang microbiome and immunological data by principal component analysis. Samples are coloured by overall sample sums, sample shapes reflect treatment group.

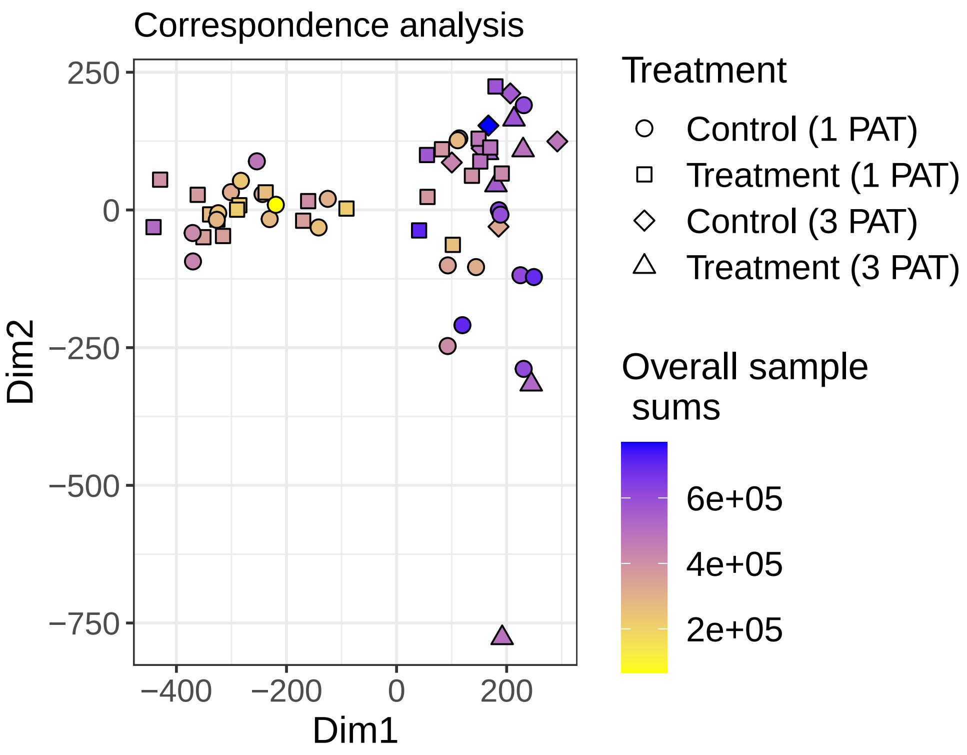

Figure S13: Sample ordination of Zhang microbiome and immunological data by correspondence analysis. Samples are coloured by overall sample sums, sample shapes reflect treatment group.

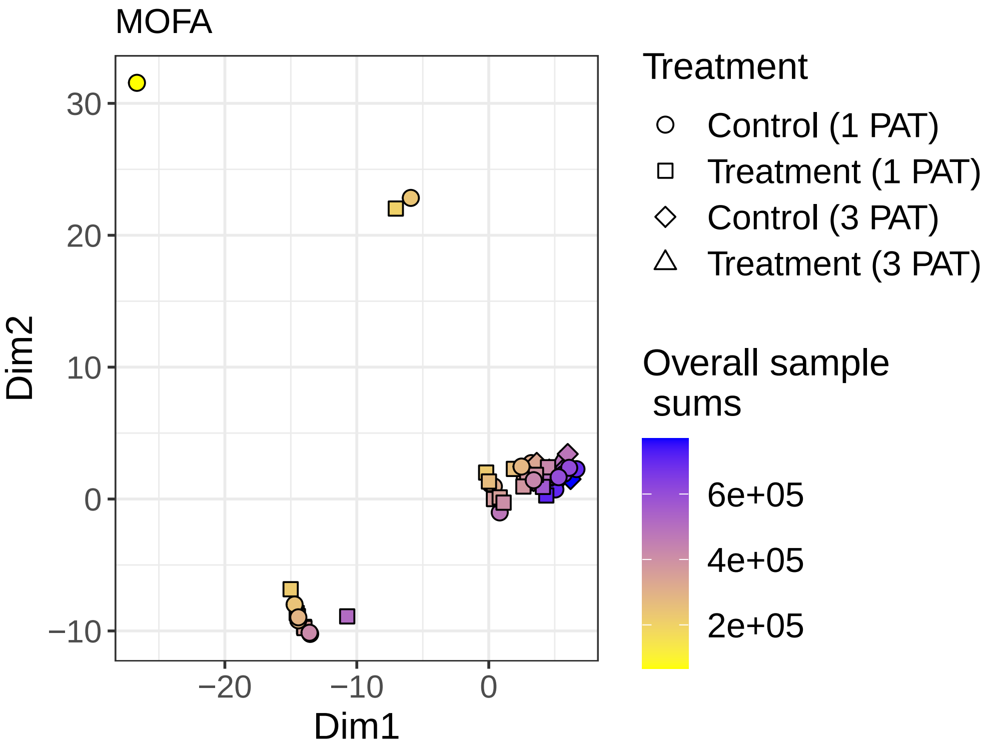

Figure S14: Sample ordination of Zhang microbiome and immunological data by MOFA. Samples are coloured by overall sample sums, sample shapes reflect treatment group.

3.2.2 Microbiome-metabolome integration

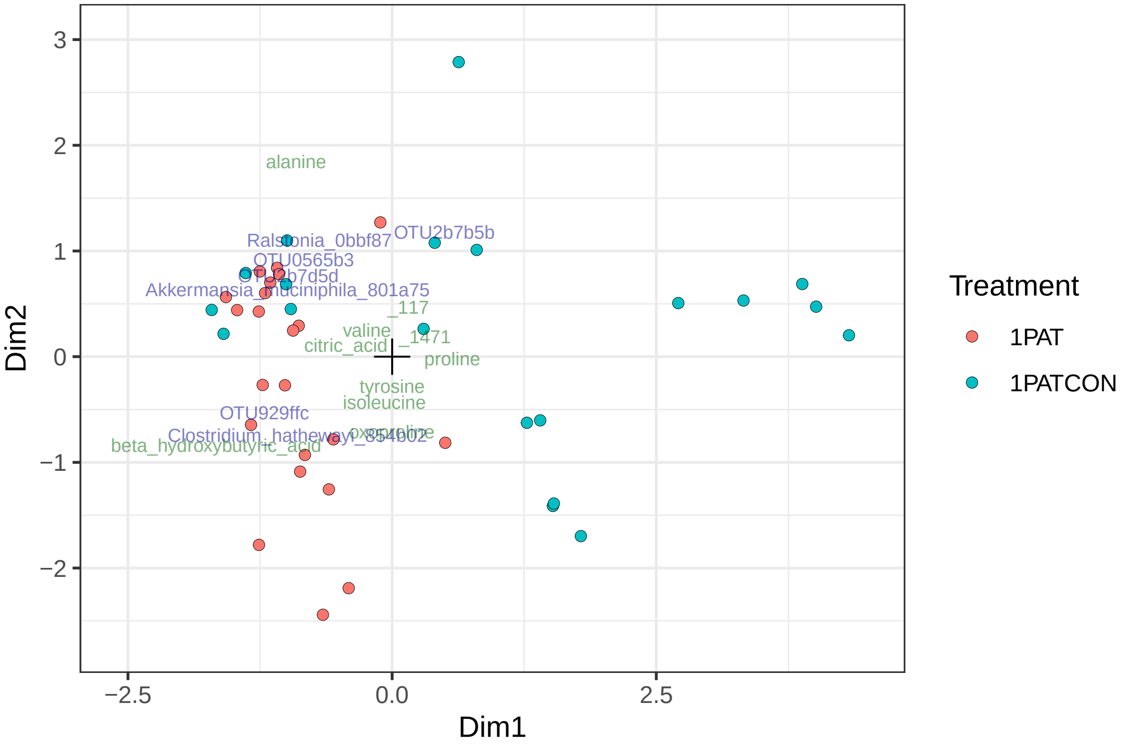

Figure S15: Unconstrained data integration of Zhang microbiome and metabolome data. The respective features are shown as blue and green labels.

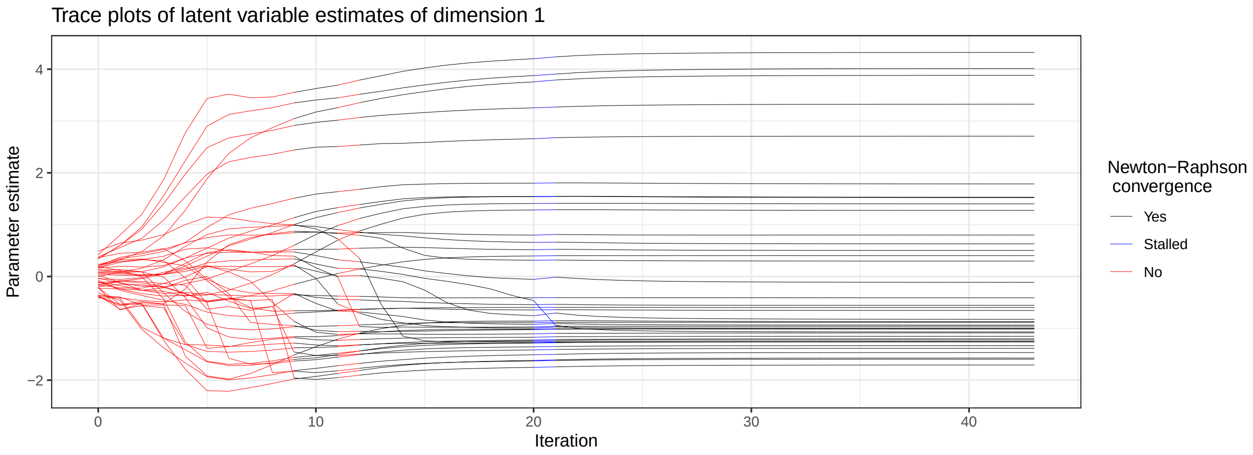

Figure S16: Convergence plot for the latent variable estimates of dimension 1 of the microbiome-metabolome integration of the Zhang data.

3.3 Gavin data

This is an observational study on T1D onset in humans. The microbiome composition was measured, as well as the human and microbial proteome from the gut.

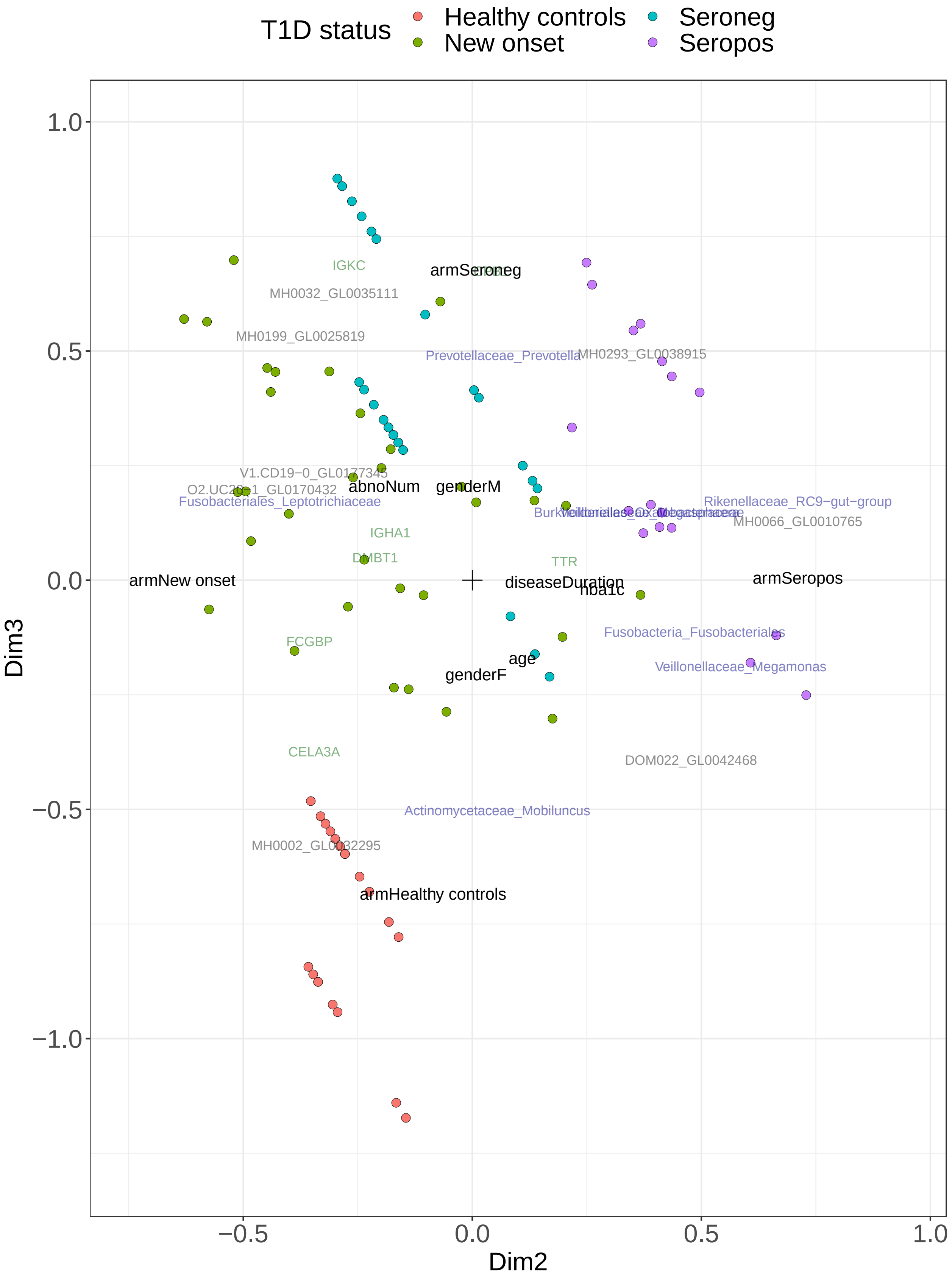

Figure S17: Constrained integration of Gavin microbiome and human and microbiological proteomics data, for the second and third dimensions. Blue labels represent taxa, red labels microbial proteins and green labels human proteins. Black labels represent components of the environmental gradient. The second dimension reveals how IGHA1 is more abundant in new onset patients than in seropositive patients, as was found by the authors too. The third dimension mainly distinguishes healthy controls from seronegative patients

4 Methods comparisons

'Data integration' is a very broad concept, and here we do not intend to give an exhaustive overview of all published methods for integration of genomics data. Instead we will focus on existing methods that provide at least either sample or feature scores such that they are (partially) comparable with our method.

4.1 Principal components analysis and correspondence analysis

Principal components analysis (PCA) can be applied after concatenating all datasets. This is probably not preferable but provides a good benchmark [23], as it yields sample scores as well as feature loadings. Also correspondence analysis [24] can be applied on concatenated matrices, but without log-ratio transform.

4.2 Canonical correlation analysis

Canonical correlation analysis (CCA) finds orthogonal pairs of linear combinations of features in $\mathbf{X}$ and $\mathbf{Y}$ with maximal correlation [25]. Sparse canonical correlation analysis (sCCA) tries to increase the interpretability by imposing sparsity on the loadings [26]. CCA does not yield unique sample scores.

4.3 Partial least squares

Partial least squares (PLS) is similar to CCA, but it finds linear combinations of variables with maximal covariance rather than correlation [27] (it might be called Canonical covariance analysis). In our case we will implement the symmetric version; i.e. we will treat matrices $\mathbf{X}$ and $\mathbf{Y}$ equally. Also a sparse version of PLS (sPLS) has been proposed [28, 29]. PLS does not yield unique sample scores.

PCA, CCA and PLS can be applied on the raw data, or on data transformed through centered log-ratio transform (clr). For count data, the zero counts are then first imputed using the *cmultRepl()* function in the *zCompositions* package [30].

4.4 MOFA

The MOFA model employs the same mean model as our data integration method [1]. Still, there are no orthogonality restrictions, and hence no biplots can be made. The parameters are estimated in a Bayesian framework, which has the advantage of natively dealing with missing values. For count data only the Poisson model is implemented. In practice this model almost never converges on datasets we presented.

4.5 JIVE

JIVE is a matrix decomposition method that decomposes standardized matrices into residuals, joint structure and view-wise structure [31]. The fitting method is also iterative, the ranks of the decomposition matrices are found through permutations. Linked matrix factorization JIVE (LMF_JIVE) is an extension to both row-wise and column-wise integration [32]. Both methods rely heavily on least squares, and require imputation to deal with missing values.

5 Simulation study

5.1 Correlation of sample scores with library sizes

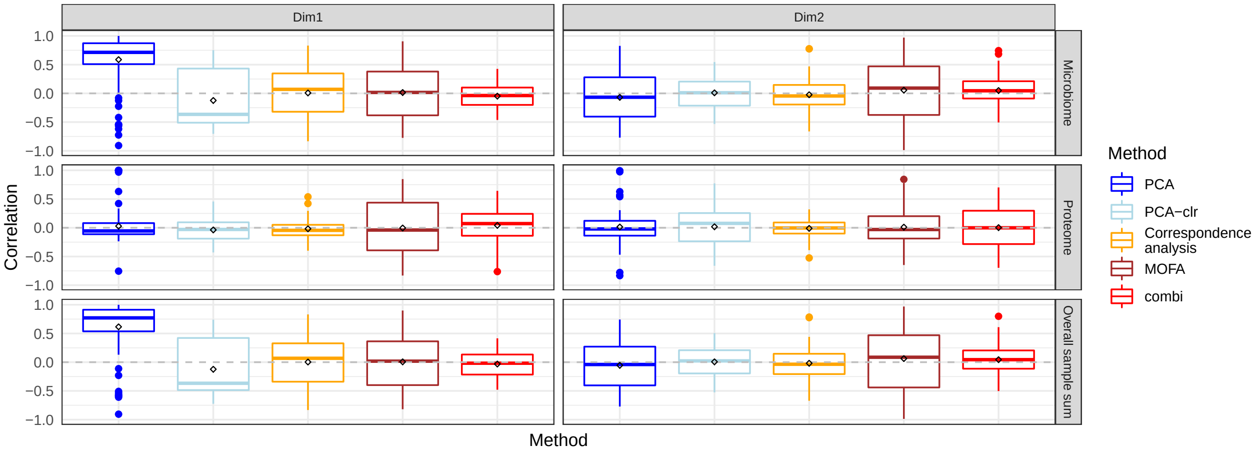

Figure S18: Boxplots of correlations with sample-wise sums (y-axis) of different datasets and overall sum (right panels) for different methods (x-axis) of different dimensions (top panels) for parametric simulation (strategy 1) based on the HMP2 microbiome and proteome datasets, without compensation.

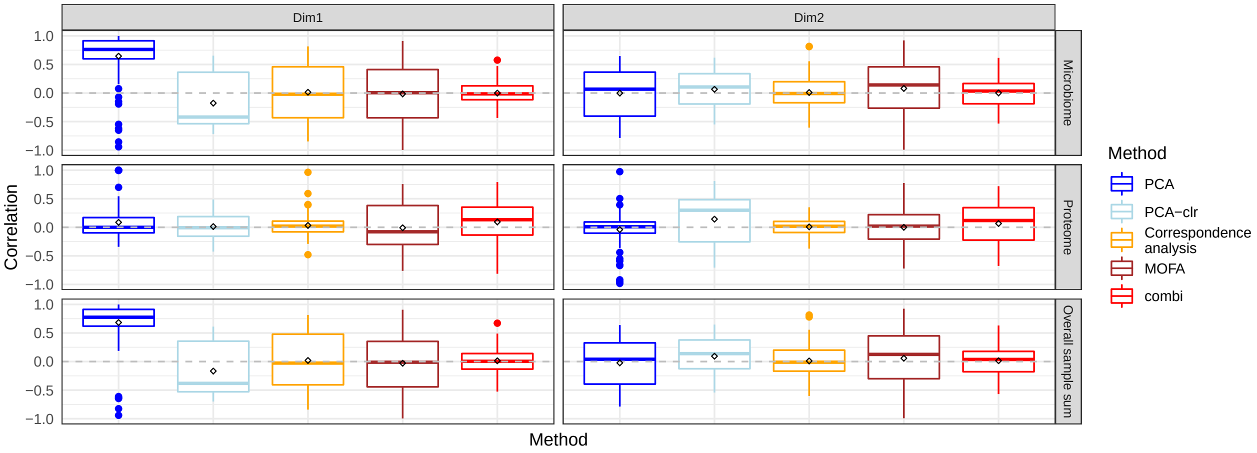

Figure S19: Boxplots of correlations with sample-wise sums (y-axis) of different datasets and overall sum (right panels) for different methods (x-axis) of different dimensions (top panels) for parametric simulation (strategy 1) based on the HMP2 microbiome and proteome datasets, with compensation.

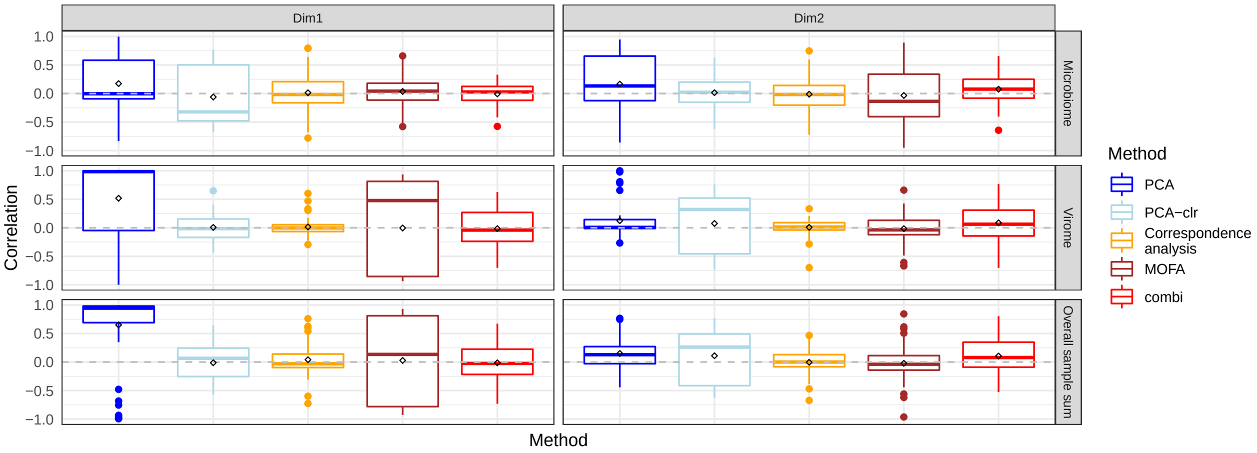

Figure S20: Boxplots of correlations with sample-wise sums (y-axis) of different datasets and overall sum (right panels) for different methods (x-axis) of different dimensions (top panels) for parametric simulation (strategy 1) based on the HMP2 microbiome and virome datasets, without compensation.

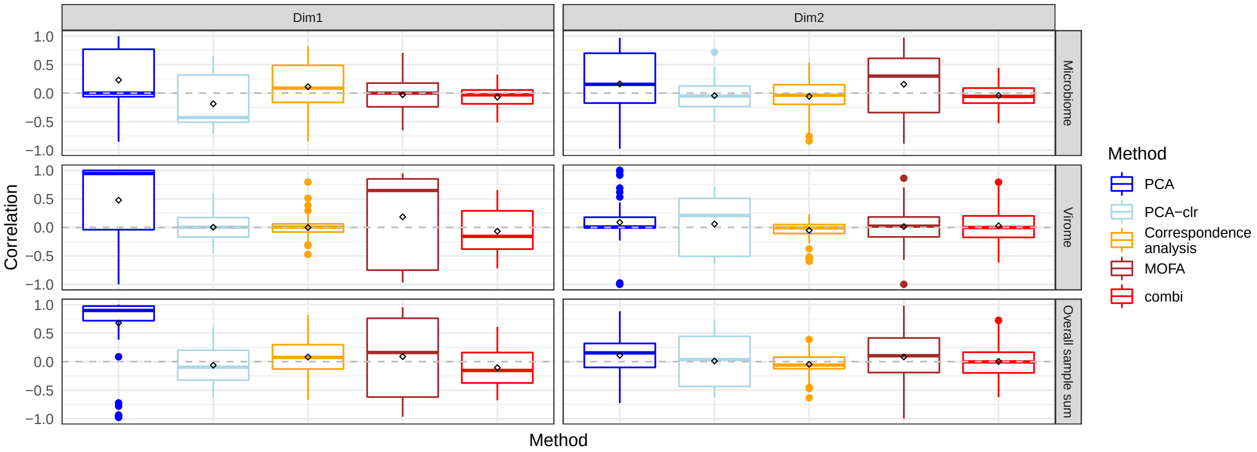

Figure S21: Boxplots of correlations with sample-wise sums (y-axis) of different datasets and overall sum (right panels) for different methods (x-axis) of different dimensions (top panels) for parametric simulation (strategy 1) based on the HMP2 microbiome and virome datasets, with compensation.

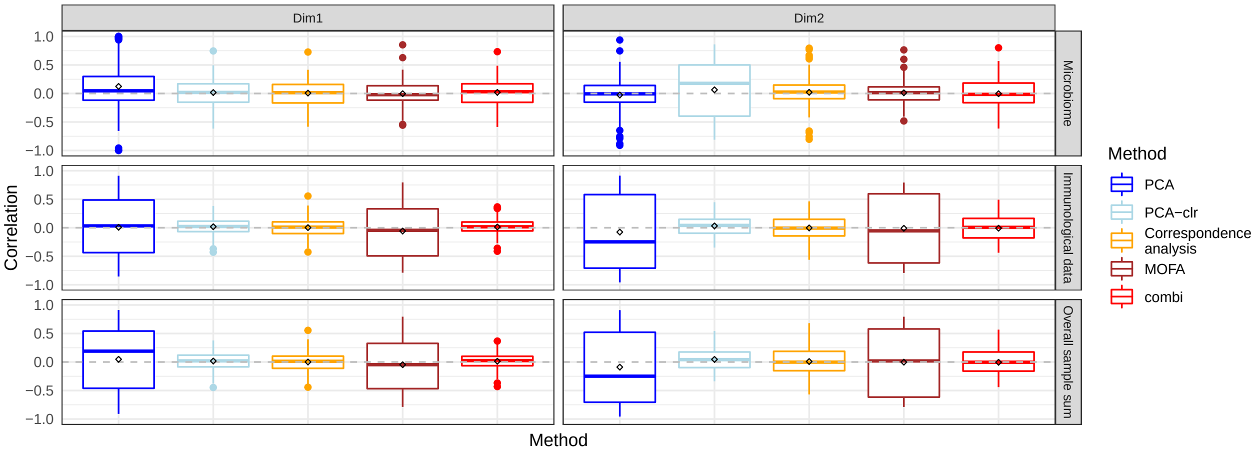

Figure S22: Boxplots of correlations with sample-wise sums (y-axis) of different datasets and overall sum (right panels) for different methods (x-axis) of different dimensions (top panels) for parametric simulation (strategy 1) based on the Zhang microbiome and immunological datasets, without compensation.

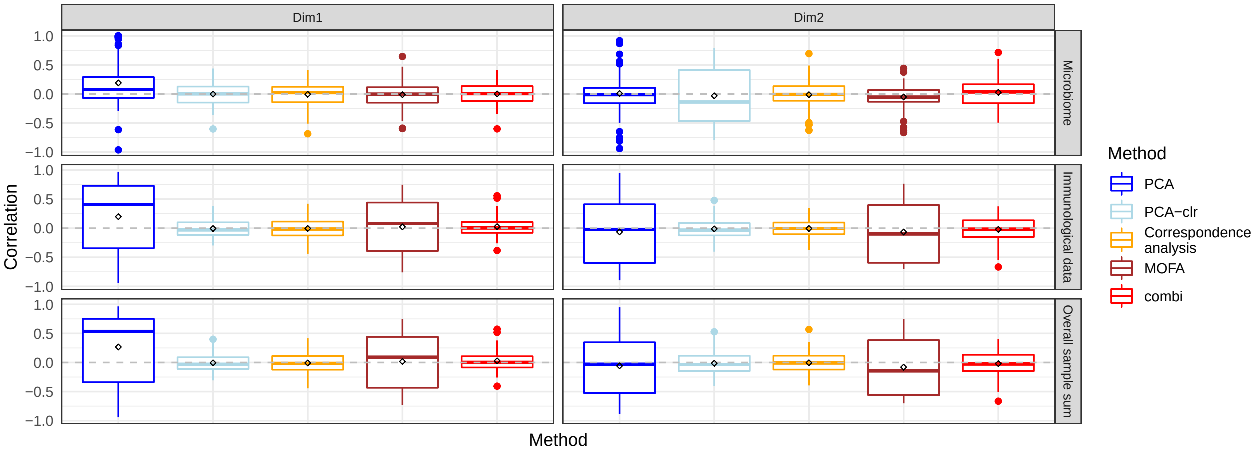

Figure S23: Boxplots of correlations with sample-wise sums (y-axis) of different datasets and overall sum (right panels) for different methods (x-axis) of different dimensions (top panels) for parametric simulation (strategy 1) based on the Zhang microbiome and immunological datasets, with compensation.

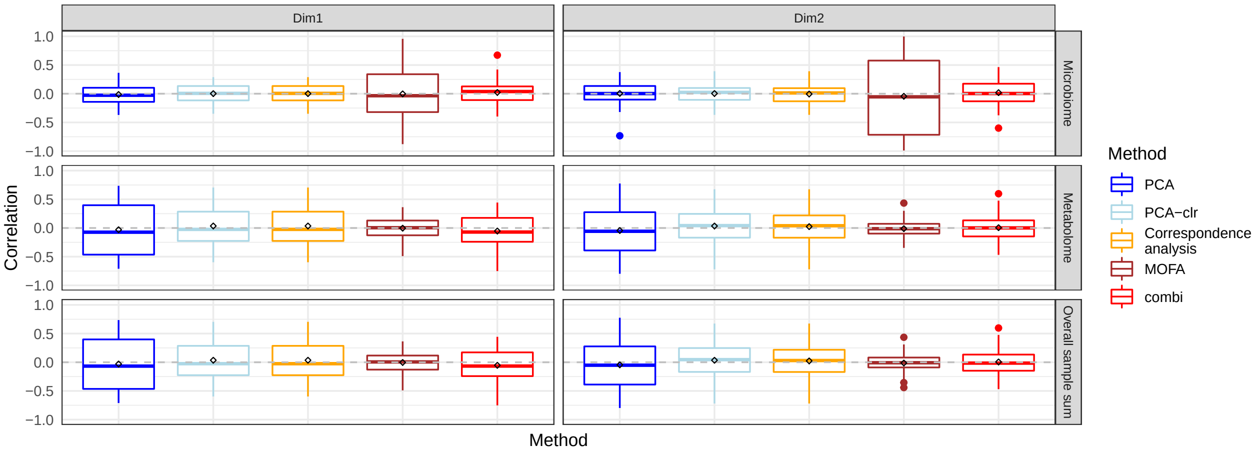

Figure S24: Boxplots of correlations with sample-wise sums (y-axis) of different datasets and overall sum (right panels) for different methods (x-axis) of different dimensions (top panels) for parametric simulation (strategy 1) based on the Zhang microbiome and metabolome datasets, without compensation.

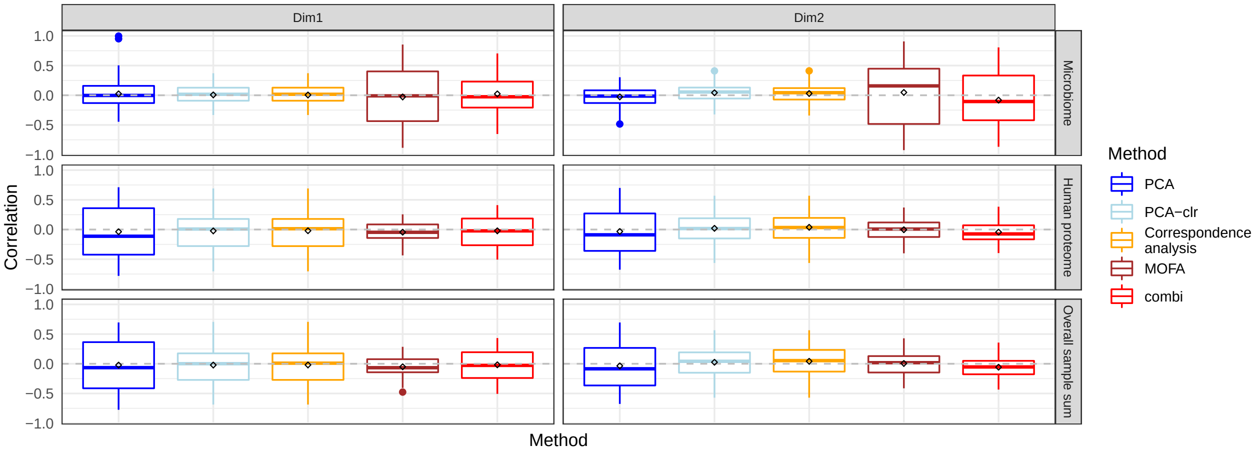

Figure S25: Boxplots of correlations with sample-wise sums (y-axis) of different datasets and overall sum (right panels) for different methods (x-axis) of different dimensions (top panels) for parametric simulation (strategy 1) based on the Gavin microbiome and human proteome datasets, without compensation.

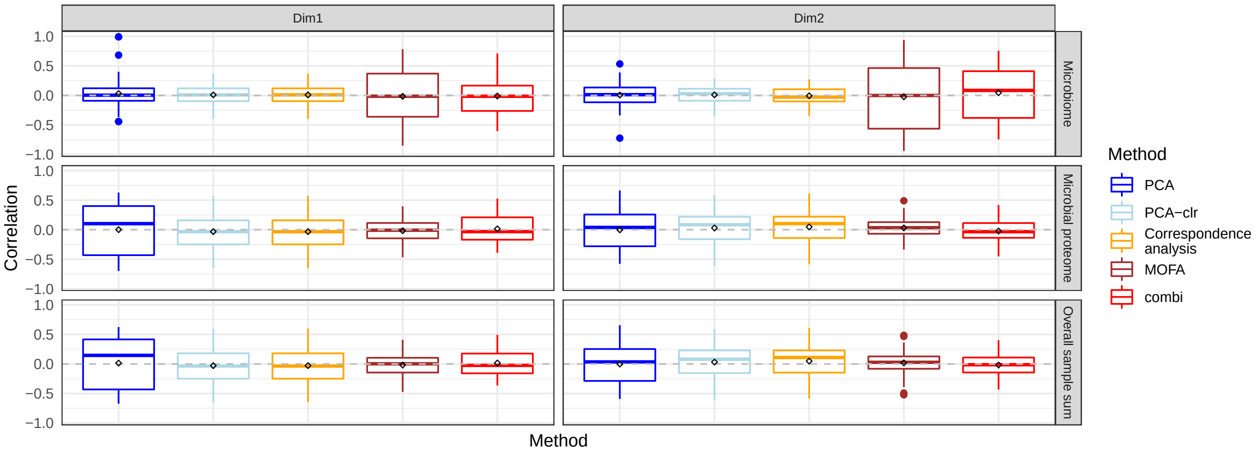

Figure S26: Boxplots of correlations with sample-wise sums (y-axis) of different datasets and overall sum (right panels) for different methods (x-axis) of different dimensions (top panels) for parametric simulation (strategy 1) based on the Gavin microbiome and microbial proteome datasets, without compensation.

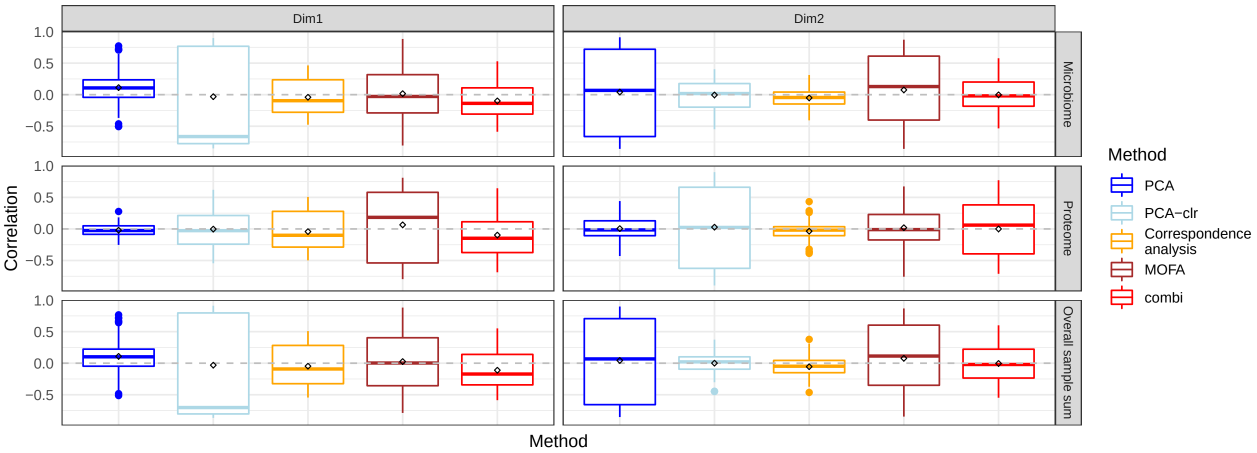

Figure S27: Boxplots of correlations with sample-wise sums (y-axis) of different datasets and overall sum (right panels) for different methods (x-axis) of different dimensions (top panels) for SimSeq data (strategy 2) generated based on the HMP2 microbiome and proteome datasets.

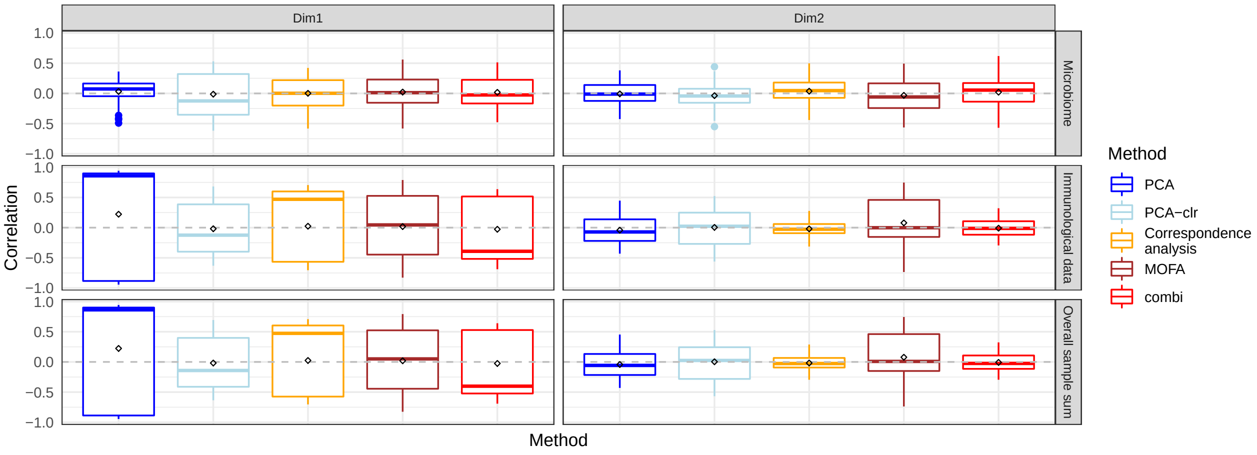

Figure S28: Boxplots of correlations with sample-wise sums (y-axis) of different datasets and overall sum (right panels) for different methods (x-axis) of different dimensions (top panels) for SimSeq data generated based on the Zhang microbiome and immunological datasets (strategy 2).

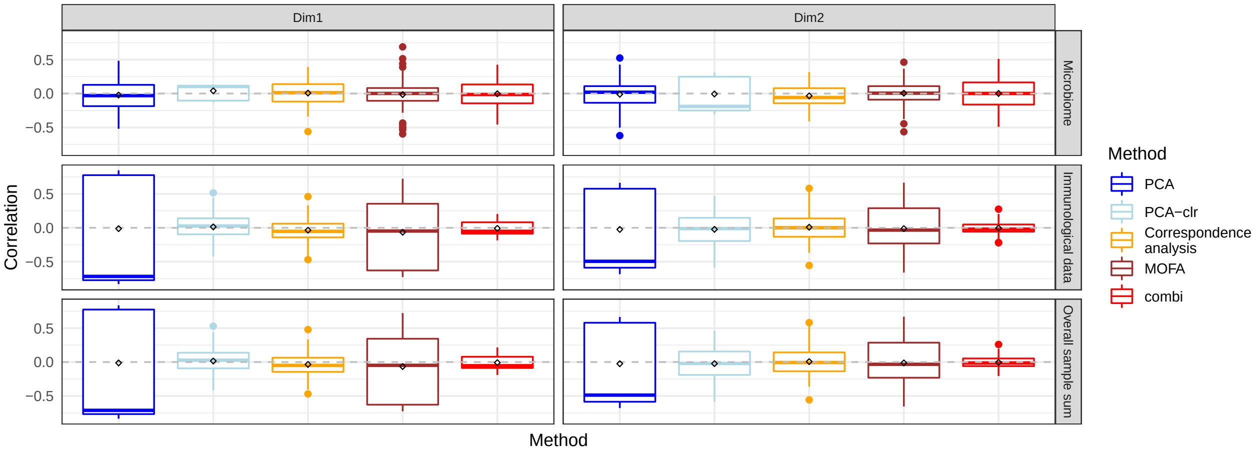

Figure S29: Boxplots of correlations with sample-wise sums (y-axis) of different datasets and overall sum (right panels) for different methods (x-axis) of different dimensions (top panels) for permuted Zhang microbiome and immunological datasets (strategy 3).

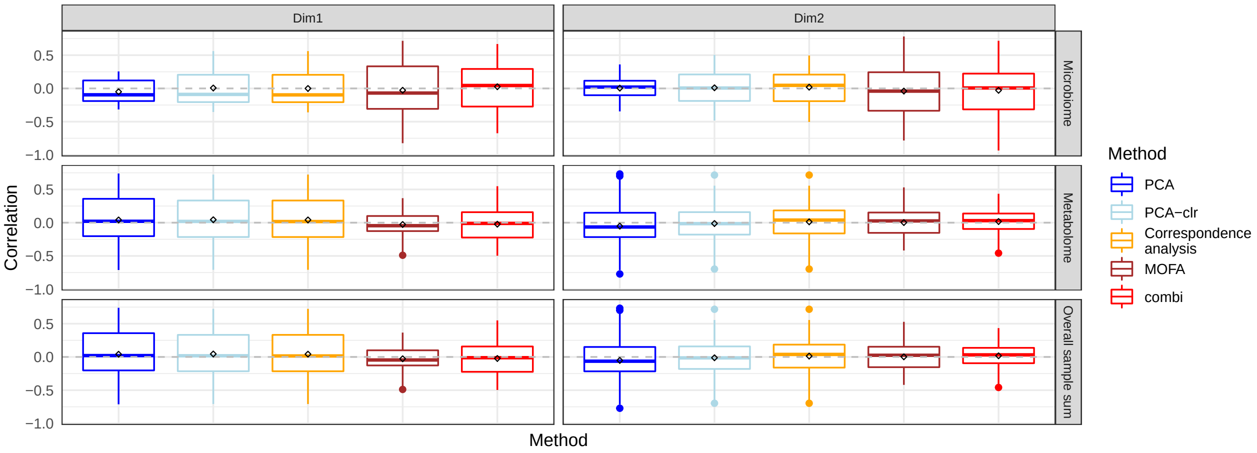

Figure S30: Boxplots of correlations with sample-wise sums (y-axis) of different datasets and overall sum (right panels) for different methods (x-axis) of different dimensions (top panels) for SimSeq data generated based on the Zhang microbiome and metabolome datasets (strategy 2).

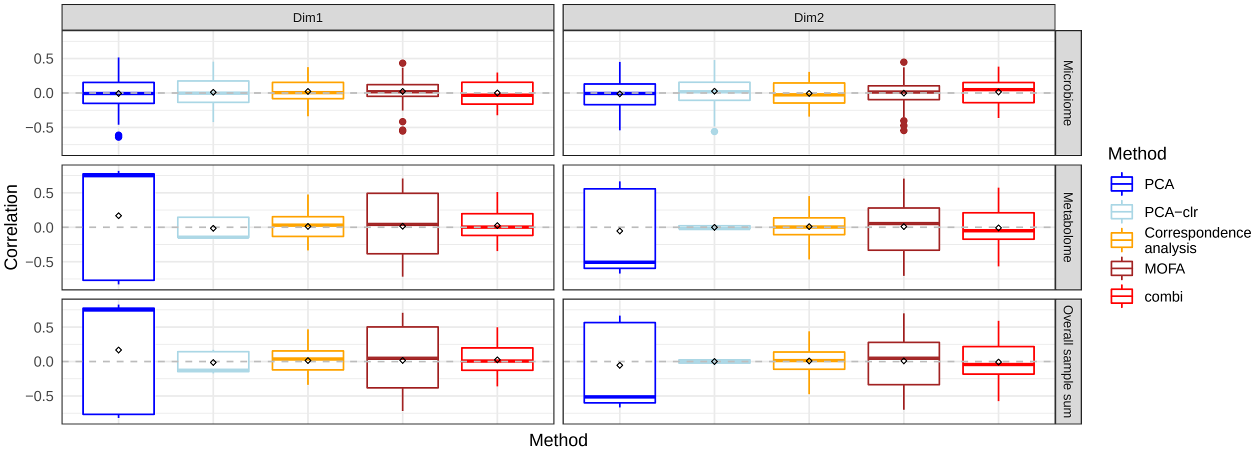

Figure S31: Boxplots of correlations with sample-wise sums (y-axis) of different datasets and overall sum (right panels) for different methods (x-axis) of different dimensions (top panels) for permuted Zhang microbiome and metabolome datasets (strategy 3).

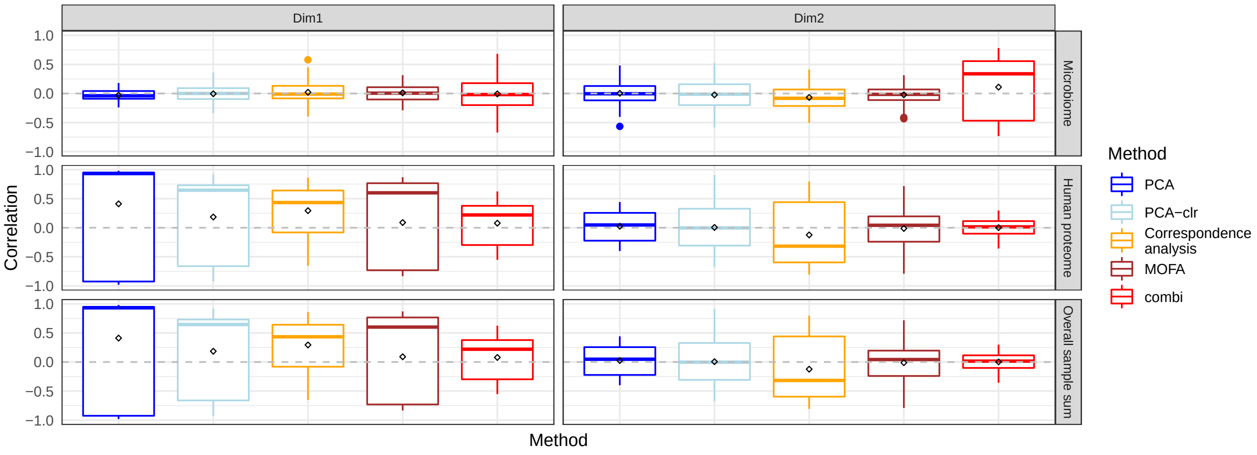

Figure S32: Boxplots of correlations with sample-wise sums (y-axis) of different datasets and overall sum (right panels) for different methods (x-axis) of different dimensions (top panels) for SimSeq data generated based on the Gavin microbiome and human protein datasets (strategy 2).

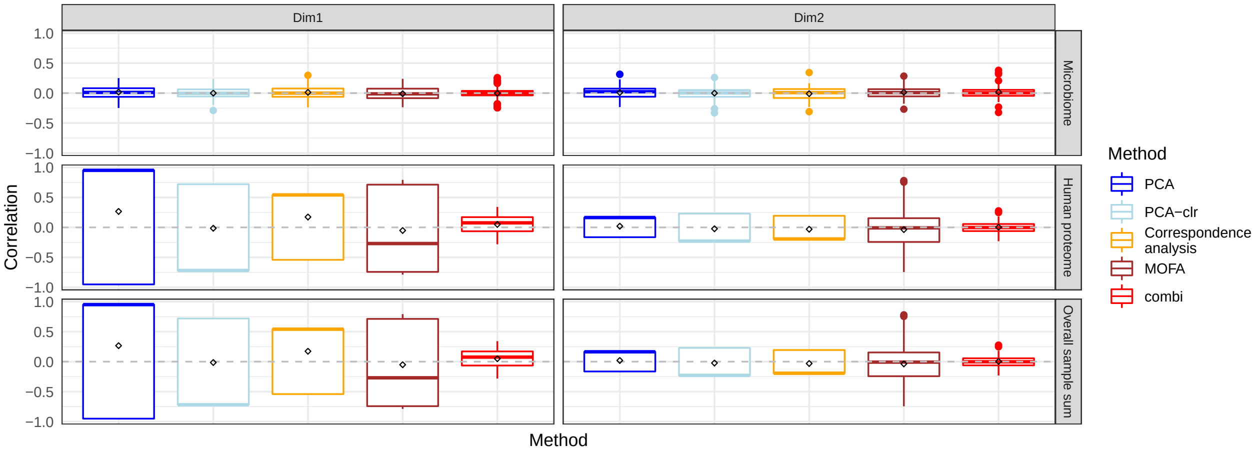

Figure S33: Boxplots of correlations with sample-wise sums (y-axis) of different datasets and overall sum (right panels) for different methods (x-axis) of different dimensions (top panels) for permuted Gavin microbiome and human protein datasets (strategy 3).

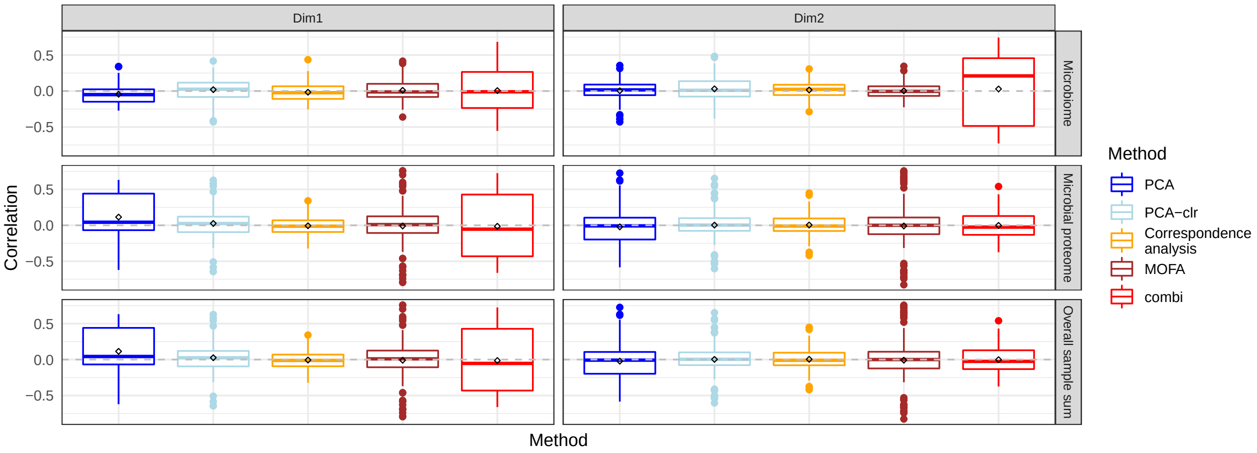

Figure S34: Boxplots of correlations with sample-wise sums (y-axis) of different datasets and overall sum (right panels) for different methods (x-axis) of different dimensions (top panels) for SimSeq data generated based on the Gavin microbiome and microbial protein datasets (strategy 2).

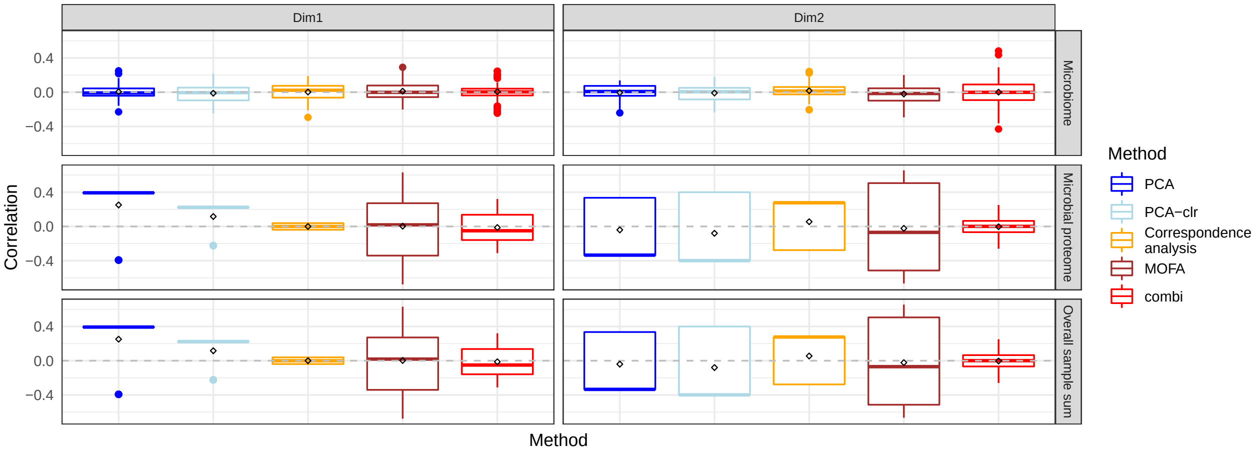

Figure S35: Boxplots of correlations with sample-wise sums (y-axis) of different datasets and overall sum (right panels) for different methods (x-axis) of different dimensions (top panels) for permuted Gavin microbiome and microbial protein datasets (strategy 3).

5.2 Identification of correlated features

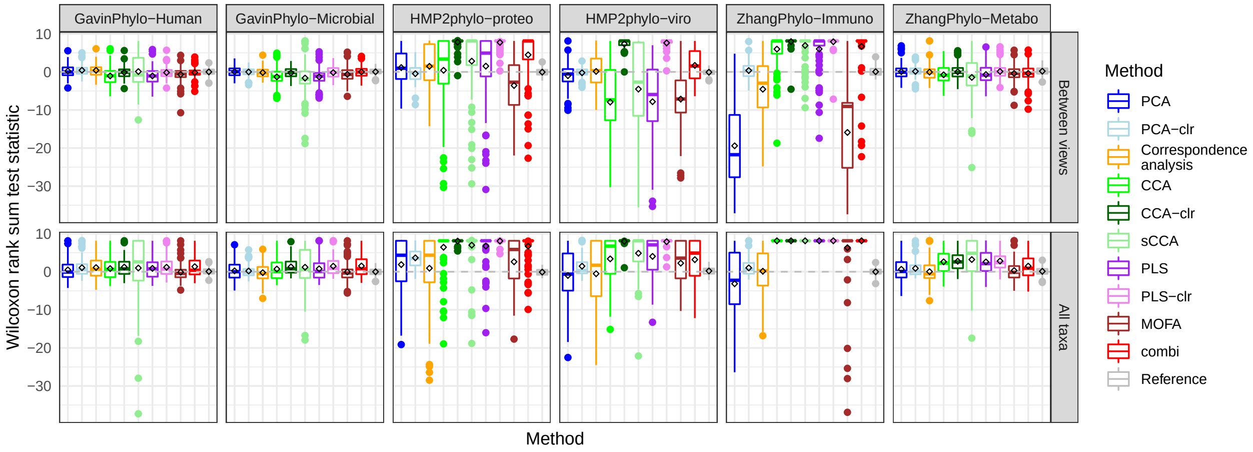

Figure S36: Boxplots of Wilcoxon rank sum test statistic quantifying correlated taxon identification for different methods (x-axis) and templates (top panels) on parametrically generated data without compensation (strategy 1).

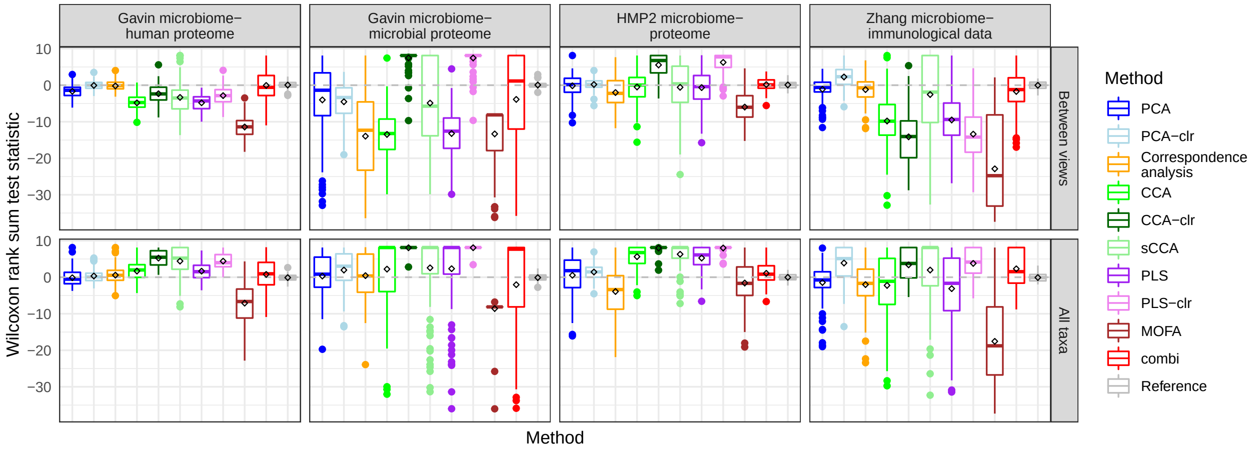

Figure S37: Boxplots of Wilcoxon rank sum test statistic quantifying correlated taxon identification for different methods (x-axis) and templates (top panels) on data generated with SimSeq (strategy 2).

5.3 Sample clustering

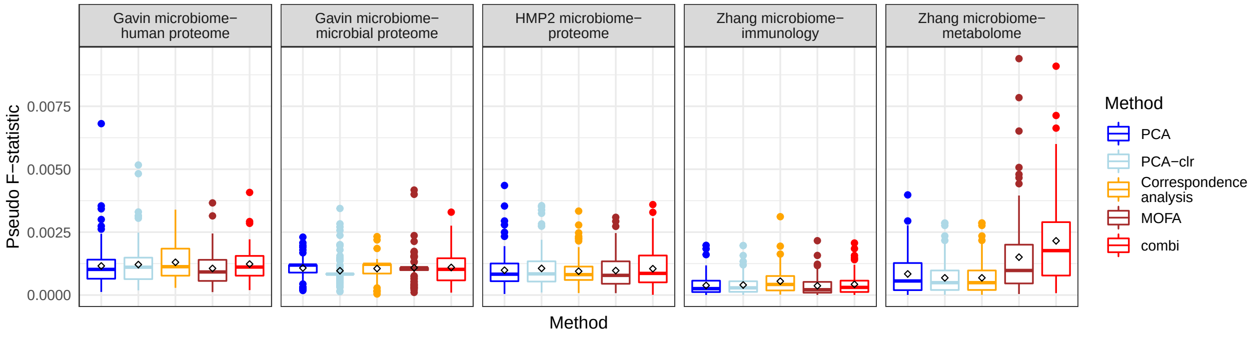

Figure S38: Boxplots of pseudo-F statistic (y-axis) quantifying sample separation for different methods (x-axis) and templates (top panels) under simulation with SimSeq (strategy 2).

6 Software

The version of R programming language and packages is shown below:

```
## R version 3.6.3 (2020-02-29)
## Platform: x86_64-pc-linux-gnu (64-bit)
## Running under: Ubuntu 18.04.4 LTS
##
## Matrix products: default
## BLAS:   /usr/lib/x86_64-linux-gnu/openblas/libblas.so.3
## LAPACK: /usr/lib/x86_64-linux-gnu/libopenblasp-r0.2.20.so
##
## locale:
##  [1] LC_CTYPE=en_US.UTF-8       LC_NUMERIC=C
##  [3] LC_TIME=en_GB.UTF-8        LC_COLLATE=en_US.UTF-8
##  [5] LC_MONETARY=en_GB.UTF-8    LC_MESSAGES=en_US.UTF-8
##  [7] LC_PAPER=en_GB.UTF-8       LC_NAME=C
##  [9] LC_ADDRESS=C               LC_TELEPHONE=C
## [11] LC_MEASUREMENT=en_GB.UTF-8 LC_IDENTIFICATION=C
##
## attached base packages:
## [1] parallel  stats     graphics  grDevices utils     datasets  methods
## [8] base
##
## other attached packages:
##  [1] RColorBrewer_1.1-2  mixOmics_6.10.9     lattice_0.20-41
##  [4] MASS_7.3-51.6       PMA_1.2.1           MOFA_1.2.0
##  [7] RCM_1.5.3           combi_0.99.15       Matrix_1.2-18
## [10] r.jive_2.1          numDeriv_2016.8-1.1 reshape2_1.4.4
## [13] ggplot2_3.3.0       phyloseq_1.30.0
##
## loaded via a namespace (and not attached):
##   [1] ggbeeswarm_0.6.0           VGAM_1.1-3
##   [3] colorspace_1.4-1           ellipsis_0.3.0
##   [5] corpcor_1.6.9              XVector_0.26.0
##   [7] GenomicRanges_1.38.0       rstudioapi_0.11
##   [9] farver_2.0.3               MatrixModels_0.4-1
##  [11] SpatioTemporal_1.1.9.1     MultiAssayExperiment_1.12.6
##  [13] ggrepel_0.8.2              RSpectra_0.16-0
##  [15] codetools_0.2-16           splines_3.6.3
##  [17] doParallel_1.0.15          knitr_1.28
##  [19] ade4_1.7-15                jsonlite_1.6.1
##  [21] cobs_1.3-4                 cluster_2.1.0
##  [23] pheatmap_1.0.12            compiler_3.6.3
##  [25] assertthat_0.2.1           limma_3.42.2
##  [27] htmltools_0.4.0            quantreg_5.55
##  [29] tools_3.6.3                igraph_1.2.5
##  [31] gtable_0.3.0               glue_1.4.1
##  [33] GenomeInfoDbData_1.2.2     dplyr_0.8.5
##  [35] Rcpp_1.0.4.6               Biobase_2.46.0
##  [37] vctrs_0.3.0                Biostrings_2.54.0
##  [39] multtest_2.42.0            gdata_2.18.0
##  [41] ape_5.3                    nlme_3.1-147
##  [43] iterators_1.0.12           xfun_0.13
```

```
## [45] stringr_1.4.0               lifecycle_0.2.0
## [47] gtools_3.8.2                nleqslv_3.3.2
## [49] zlibbioc_1.32.0             zoo_1.8-8
## [51] scales_1.1.1                SummarizedExperiment_1.16.1
## [53] biomformat_1.14.0           rhdf5_2.30.1
## [55] SparseM_1.78                yaml_2.2.1
## [57] quantmod_0.4.17             curl_4.3
## [59] gridExtra_2.3               reticulate_1.15
## [61] stringi_1.4.6               highr_0.8
## [63] S4Vectors_0.24.4            tseries_0.10-47
## [65] corrplot_0.84               foreach_1.5.0
## [67] permute_0.9-5               BB_2019.10-1
## [69] TTR_0.23-6                  caTools_1.18.0
## [71] BiocGenerics_0.32.0         BiocParallel_1.20.1
## [73] GenomeInfoDb_1.22.1         rlang_0.4.6
## [75] pkgconfig_2.0.3             matrixStats_0.56.0
## [77] bitops_1.0-6                evaluate_0.14
## [79] purrr_0.3.4                 tensor_1.5
## [81] Rhdf5lib_1.8.0              labeling_0.3
## [83] cowplot_1.0.0               tidyselect_1.1.0
## [85] plyr_1.8.6                  magrittr_1.5
## [87] R6_2.4.1                    IRanges_2.20.2
## [89] gplots_3.0.3                DelayedArray_0.12.3
## [91] pillar_1.4.4                withr_2.2.0
## [93] mgcv_1.8-31                 xts_0.12-0
## [95] survival_3.1-12             abind_1.4-5
## [97] RCurl_1.98-1.2              tibble_3.0.1
## [99] crayon_1.3.4                rARPACK_0.11-0
## [101] KernSmooth_2.23-17         ellipse_0.4.1
## [103] alabama_2015.3-1           rmarkdown_2.1
## [105] grid_3.6.3                 data.table_1.12.8
## [107] vegan_2.5-6                digest_0.6.25
## [109] tidyr_1.0.3                stats4_3.6.3
## [111] munsell_0.5.0              beeswarm_0.2.3
## [113] vipor_0.4.5                quadprog_1.5-8
```

References

1. Argelaguet, R, Velten, B, et al. Multi-Omics Factor Analysis—a framework for unsupervised integration of multi-omics data sets. Molecular Systems Biology 2018;14.
2. Billheimer, D, Guttorp, P, and Fagan, WF. Statistical Interpretation of Species Composition. Journal of the American Statistical Association 2001;96:1205 –1214.
3. Firth, D. Bias Reduction of Maximum Likelihood Estimates. Biometrika 1993;80:27 –38.
4. Kosmidis, I and Firth, D. Bias reduction in exponential family nonlinear models. Biometrika 2009;96:793 –804.
5. Lund, S, Nettleton, D, et al. Detecting Differential Expression in RNA-sequence Data Using Quasi-likelihood with Shrunken Dispersion Estimates. 2012;11.
6. Hawinkel, S, Kerckhof, F-M, et al. A unified framework for unconstrained and constrained ordination of microbiome read count data. PLOS ONE 2019;14:1 –20.
7. van den Wollenberg, AL. Redundancy analysis an alternative for canonical correlation analysis. Psychometrika 1977;42:207 –219.
8. Anders, S and Huber, W. Differential expression analysis for sequence count data. Genome Biol 2010;11:R106–R106.

9. Robinson, MD and Smyth, GK. Moderated statistical tests for assessing differences in tag abundance. Bioinformatics 2007;23:2881–2887.
10. Marioni, JC, Mason, CE, et al. RNA-seq: An assessment of technical reproducibility and comparison with gene expression arrays. Genome Res 2008;18:1509–1517.
11. Ritchie, ME, Phipson, B, et al. Limma powers differential expression analyses for RNA-sequencing and microarray studies. Nucleic Acids Res. 2015;43:e47.
12. Smyth, GK. Linear models and empirical Bayes methods for assessing differential expression in microarray experiments. STAT. APPL. GENET. MOL. BIOL 2004;3.
13. Hampel, FR, Ronchetti, EM, et al. Robust Statistics: The Approach Based on Influence Functions. Vol. 07. John Wiley & Sons, Inc., 2011.
14. Aitchison, J. The Statistical Analysis of Compositional Data. Journal of the Royal Statistical Society. Series B (Methodological) 1982;44:139 –177.
15. Gloor, GB and Reid, G. Compositional analysis: A valid approach to analyze microbiome high-throughput sequencing data. Can. J. Microbiol. 2016;62:692 –703.
16. Hawinkel, S, Kerckhof, F-M, et al. A unified framework for unconstrained and constrained ordination of microbiome read count data. bioRxiv 2018.
17. Gabriel, KR. The biplot graphic display of matrices with application to principal component analysis. Biometrika 1971;58:453 –467.
18. Xia, F, Chen, J, et al. A Logistic Normal Multinomial Regression Model for Microbiome Compositional Data Analysis. Biometrics 2013;69:1053 –1063.
19. Aitchison, J and Greenacre, M. Biplots of Compositional Data. Journal of the Royal Statistical Society. Series C (Applied Statistics) 2002;51:375 –392.
20. Shannon, CE. A Mathematical Theory of Communication. Bell System Technical Journal 1948;27:379 –423.
21. O'Brien, JJ, O'Connell, JD, et al. Compositional Proteomics: Effects of Spatial Constraints on Protein Quantification Utilizing Isobaric Tags. J. Proteome Res. 2018;17:590 –599.
22. Zhang, X-S, Li, J, et al. Antibiotic-induced acceleration of type 1 diabetes alters maturation of innate intestinal immunity. eLife 2018;7:e37816.
23. Westerhuis, JA, Kourti, T, and MacGregor, JF. Analysis of multiblock and hierarchical PCA and PLS models. J. Chemom. 1998;12:301 –321.
24. Benzecri, J. L'analyse des données. Population 1975;30:1190.
25. Hotelling, H. The most predictable criterion. Journal of Educational Psychology 1935;26:139 –142.
26. Wilms, I and Croux, C. Robust sparse canonical correlation analysis. BMC Syst Biol 2016;10:72 –72.
27. Wold, S, Ruhe, A, et al. The Collinearity Problem in Linear Regression. The Partial Least Squares (PLS) Approach to Generalized Inverses. SIAM Journal on Scientific and Statistical Computing 1984;5:735 –743.
28. Tenenhaus, A, Philippe, C, et al. Variable selection for generalized canonical correlation analysis. Biostatistics 2014;15:569 –583.
29. Cao, K-AL t., Debra, R, et al. A Sparse PLS for Variable Selection when Integrating Omics Data. sagmb 2008;7.
30. Palarea-Albaladejo, J and Martin-Fernandez, JA. ZCompositions – R package for multivariate imputation of left-censored data under a compositional approach. Chemom. Intell. Lab. Syst. 2015;143:85 –96.
31. Lock, EF, Hoadley, KA, et al. Joint and individual variation explained (jive) for integrated analysis of multiple data types. Ann Appl Stat 2013;7:523 –542.
32. O'Connell, MJ and Lock, EF. Linked matrix factorization. Biometrics 2018;0.
